# Bclaf1 promotes angiogenesis by regulating HIF-1α transcription in hepatocellular carcinoma

**DOI:** 10.1038/s41388-018-0552-1

**Published:** 2018-10-26

**Authors:** Ying Wen, Xueqiong Zhou, Meiting Lu, Meiling He, Ye Tian, Lixia Liu, Mengnan Wang, Wenchong Tan, Yaotang Deng, Xushan Yang, Matthias P. Mayer, Fei Zou, Xuemei Chen

**Affiliations:** 1 Department of Occupational Health and Medicine, Guangdong Provincial Key Laboratory of Tropical Disease Research, School of Public Health, Southern Medical University, 1838 Guangzhou Road North, Guangzhou, 510515 China; 2 Center for Molecular Biology of Heidelberg University (ZMBH), DKFZ-ZMBH-Alliance, Im Neuenheimer Feld 282, 69120 Heidelberg, Germany

**Keywords:** Prognostic markers, Cancer microenvironment

## Abstract

The development of hepatocellular carcinomas (HCC) depends on their local microenvironment and the induction of neovascularization is a decisive step in tumor progression, since the growth of solid tumors is limited by nutrient and oxygen supply. Hypoxia is the critical factor that induces transcription of the hypoxia inducible factor-1α (HIF-1α) encoding gene HIF1A and HIF-1α protein accumulation to promote angiogenesis. However, the basis for the transcriptional regulation of HIF1A expression in HCC is still unclear. Here, we show that Bclaf1 levels are highly correlated with HIF-1α levels in HCC tissues, and that knockdown of Bclaf1 in HCC cell lines significantly reduces hypoxia-induced HIF1A expression. Furthermore, we found that Bclaf1 promotes HIF1A transcription via its bZIP domain, leading subsequently to increased transcription of the HIF-1α downstream targets VEGFA, TGFB, and EPO that in turn promote HCC-associated angiogenesis and thus survival and thriving of HCC cells. Moreover, we demonstrate that HIF-1α levels and microvessel density decrease after the shRNA-mediated Bclaf1 knockdown in xenograft tumors. Finally, we found that Bclaf1 levels increase in hypoxia in a HIF-1α dependent manner. Therefore, our study identifies Bclaf1 as a novel positive regulator of HIF-1α in the hypoxic microenvironment, providing new incentives for promoting Bcalf1 as a potential therapeutic target for an anti-HCC strategy.

## Introduction

Hepatocellular carcinoma (HCC) is the 2nd leading cause of global cancer morbidity and mortality [1], and brings about 750,000 new patients and 695,000 deaths per year [2]. Therefore, the elucidation of the molecular mechanisms of HCC pathogenesis and progression would provide a strong theoretical basis for targeted therapies and drug design.

The local microenvironment is essential for the development and progression of the HCC. Due to insufficient blood supply, a prominent feature of solid tumors like HCC is hypoxia that induces cancer cells to change their signaling pathways and metabolic processes to adapt to the hypoxic challenge. Hypoxia inducible factor-1α (HIF-1α), a well-defined hypoxia responsive factor, activates diverse pathways that regulate cellular metabolism, angiogenesis, proliferation, and drug resistance [3, 4]. In a normal oxygen environment, HIF-1α is hydroxylated and rapidly degraded by the ubiquitin-proteasome pathway. In hypoxic conditions, HIF-1α is no longer degraded but translocates into the nucleus and binds HIF-1β to activate the transcription of downstream genes [4]. It has been shown that the up-regulation of HIF-1α activity promotes tumor-associated angiogenesis, and hence the survival and proliferation of tumor cells in solid tumors [5, 6]. HCC is a hypervascularized tumor and inhibiting angiogenesis is envisioned to be a potential strategy to control HCC [7]. Consequently, it is crucial to elucidate the regulatory pathway leading to angiogenesis in the HCC context, starting at HIF-1α.

To date, it is well established that HIF-1α protein levels are mainly controlled by post-translational modifications and degradation, as mentioned above, but there has been increasing awareness that under hypoxic conditions, HIF-1α amounts can also be regulated at the level of mRNA by transcription factors [8–10], microRNAs, and RNA-binding proteins [11, 12] through growth factor signaling pathways and other control circuits [13, 14]. We show here that Bcl-2-associated transcription factor 1 (Bclaf1) is one of the factors that regulate HIF1A transcription.

Bclaf1 was originally identified as a protein interacting with anti-apoptotic members of the Bcl2 family [15, 16]. Over the years, it has been reported to be involved in various biological processes [17], including T-cell activation, lung development [18], control of the lytic infection program [19], DNA damage and repair [20, 21], and even post-transcriptional events like pre-mRNA splicing [22] and mRNA processing [23]. Although most of the studies suggest that Bclaf1 is a tumor suppressor [15], a recent study reported that Bclaf1 regulates the tumorigenesis of colon cancer cells [24], implying that there is a potential correlation between Bclaf1 and tumor progression. The prominent features of the Bclaf1 structure are the bZIP and the Myb DNA-binding domains, which are essential for Bclaf1 function in transcriptional regulation. However, little is known about their roles under hypoxic conditions [17].

Interestingly, our RNA-sequencing result revealed that depletion of Bclaf1 associates with the inhibition of the angiogenesis pathway and down-regulation of HIF1A mRNA levels. Thus, we further explored the effect of Bclaf1 on HIF-1α transcription and protein levels and the biological significance of Bclaf1 in HCC angiogenesis and tumor progression under hypoxic conditions.

## Results

### Bclaf1 knockdown inhibits hypoxia and angiogenesis pathways

**Fig. 1:**
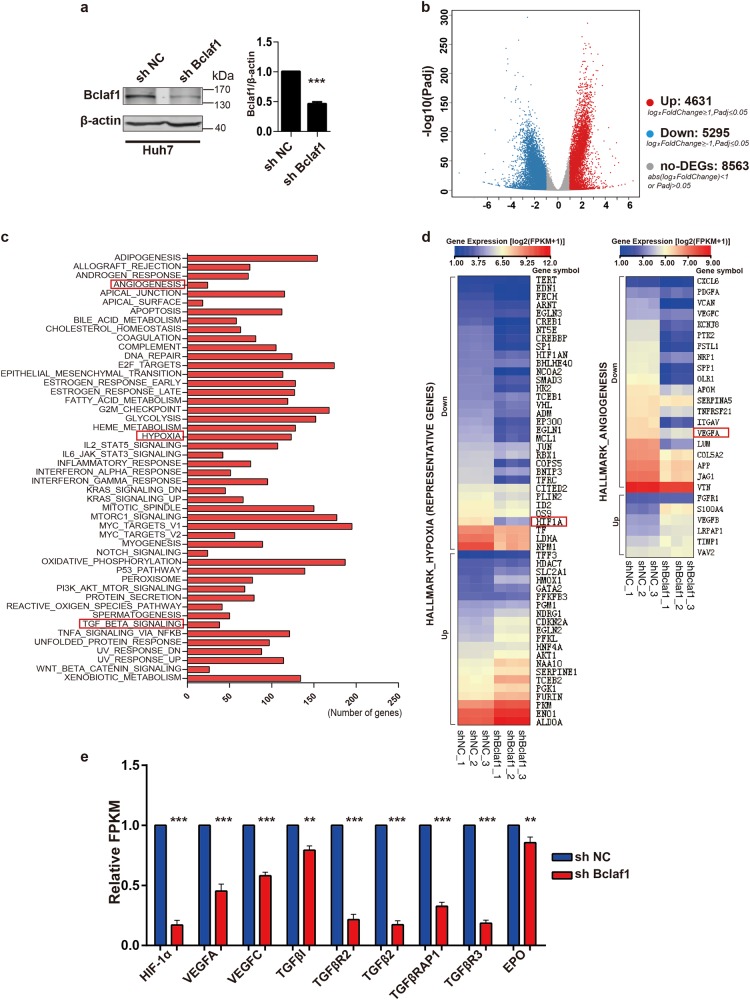
RNAseq results reveal Bclaf1 knockdown inhibits hypoxia and angiogenesis pathway in Huh7 cells. mRNA was isolated from Huh7 cells stably transfected with shNC or shBclaf1 and analyzed by RNAseq. **a** The residual level of Bclaf1 protein in Huh7 cells transfected with shRNA targeting BCLAF1 mRNA as compared to the shNC cells was evaluated by immunoblotting (mean ± SD of three independent experiments. ***p < 0.001). **b** Volcano plot of differentially expressed genes between shNC and shBCLAF1 Huh7 cells as determined by RNAseq. **c** Gene Set Enrichment Analysis (GSEA) to categorize the pathways that are significantly altered upon Bclaf1 knockdown. Hypoxia, angiogenesis, and TGF-β signaling are highlighted. **d** Differentially regulated angiogenesis and hypoxia signaling-related genes were shown in a heat map by MeV 4.0, data was presented as log_2_(FPKM + 1). **e** Validation of identified hypoxia and angiogenesis related genes through FPKM (Reads Per Kilobase of exon model per Million mapped reads, FPKM ≥ 1) normalized to the shNC control of three independent sample. **p < 0.01, ***p < 0.001 vs. control

To characterize Bclaf1-dependent global changes in the transcriptome, we conducted a genome-wide RNA sequencing (RNAseq) analysis. Total RNAs of Huh7 cells transfected with a BCLAF1 targeting shRNA (shBclaf1) or a control shRNA (shNC) were isolated and subjected to sequencing. The Bclaf1 protein level in the shBclaf1 transfected cells was reduced to 50% of the level in shNC Huh7 cells as determined by immunoblotting prior to RNAseq (Fig. 1a). Based on the significance criterion (*p*-value), a total of 9926 transcripts were significantly changed in shBclaf1 cells as compared to shNC cells (Fig. 1b). Subsequent Gene Set Enrichment Analysis (GSEA) revealed that the enriched categories include hypoxia, angiogenesis, and TGF-β signaling (Fig. 1c). Representative genes related to hypoxia and angiogenesis pathways are displayed as a heat map (Fig. 1d). Interestingly, the expression levels of HIF1A and its downstream angiogenic target genes VEGFA, VEGFC, TGFB2, TGFBR2, TGFBR3, TGFBI, TGFBRAP1, and EPO, which are known to be HCC angiogenesis-related [25, 26], were significantly down-regulated in response to Bclaf1 knockdown (Fig. 1e). These data suggested that Bclaf1 is involved in angiogenesis in the hypoxic microenvironment of tumors. Since HIF-1α is the oxygen sensor that controls the expression of multiple target genes implicated in angiogenesis, including VEGF, TGFB, EPO [6, 26], we then focused on the role of Bclaf1 in modulating angiogenesis and HIF1A expression under hypoxia.

### Hypoxia enhances Bclaf1-mediated growth promotion and angiogenesis

**Fig. 2:**
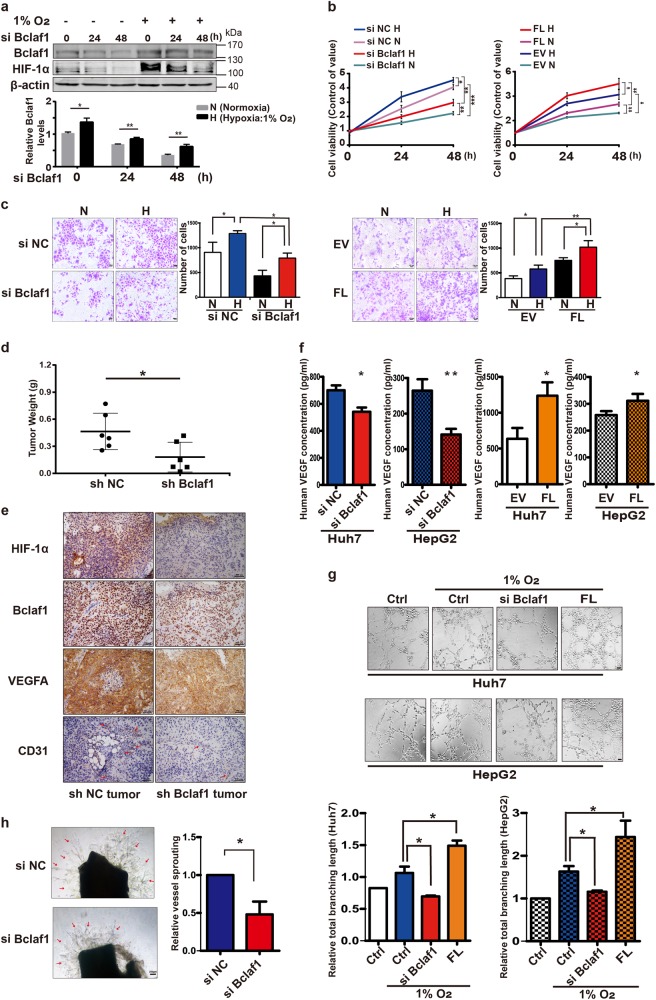
Bclaf1 promotes the growth of HCC cells and angiogenesis in hypoxia. **a** Western blot analysis of Huh7 cells cultured in 1% O_2_ or 20% O_2_ for the indicated hours. Gel-Pro software was used to determine the relative intensities of Bclaf1 and HIF-1α bands. **b**, **c** Hypoxia promotes cell viability and siBCLAF1 compromises cell viability. Huh7 cells were transfected with siBCLAF1 and siNC as control, or a BCLAF1 expressing plasmid (FL) and an empty plasmid (EV) as control. 24 h post-transfection, the cells were cultured under hypoxic conditions or left under normal O_2_ conditions for 24 h. Cell proliferation was assessed using the CCK-8 assay (**b**) and cell numbers were determined after crystal violet staining by wide field microscopy (**c**). **d**, **e** Bclaf1 affects tumor growth and VEGFA secretion in a mouse xenograft model. **d** Stably shBCLAF1 and shNC transfected Huh7 cells were implanted into nude mice, the xenograft tumors were surgically extracted 7 weeks after implantation, and tumor weights were measured. Each point represents the mean weight ± SD, *n* = 6, *p < 0.05. **e** Immunohistochemistry (200×) of HIF-1α, VEGFA, CD31, and Bclaf1 in the tumor tissues of orthotopic hepatocarcinoma xenografts. Red arrows point to CD31. **f** Bclaf1 affects VEGFA secretion in HCC in vitro. HCC cells were transfected with siBCLAF1 or with a BCLAF1 overexpressing plasmid or with the respective controls (siNC, EV) and exposed to hypoxia for 48 h before VEGFA concentrations were determined by ELISA in the culture supernatants. Data shown represent the mean ± SD (*n* = 3) *p < 0.05, **p < 0.01. **g** Bclaf1 promotes tube formation of endothelial cells. HUVECs were plated on Matrigel-coated plates at a density of 1.5 × 10^5^ cells/well and incubated for 24 h before the cell culture supernatant of siBCLAF1 or BCLAF1 overexpression plasmid transfected and hypoxia treated HCC cells was added. Images were taken 12 h after addition of the supernatant (200×). Tube networks were quantified using the ImageJ software and expressed as branching length. **h** Bclaf1 promotes sprouting of vessels from Aortic rings. Aortic segments were harvested from C57BL/6 mice. Aortic segments in Matrigel were treated for 7 days with the culture supernatant of siBCLAF1 or Bclaf1 overexpression plasmid transfected and hypoxia treated Huh7 cells (*n* = 3 per group). All data represent mean ± SD of three independent experiments. *p < 0.05, **p < 0.01, ***p < 0.001 vs. control

Since hypoxia stimulates HCC growth and is one of the critical microenvironmental factors in HCC [27, 28], we aimed to elucidate the biologic role of Bclaf1 in HCC cells after exposure to hypoxia (1% oxygen). Exposure of HCC cells to hypoxic conditions significantly increased Bclaf1 levels (Fig. 2a) and simultaneous application of hypoxia and down-regulation of Bclaf1 by siRNA transfection reduced the growth rate of Huh7 cells in comparison to siRNA control cells under hypoxia. In contrast, transient transfection with a plasmid expressing the Bclaf1-encoding gene (FL) dramatically enhanced proliferative capacity of Huh7 cells (Fig. 2b, c). We validated the oncogenic role of Bclaf1 in vivo. Bclaf1 depleted Huh7 cells (with a stably integrated BCLAF1-shRNA expression construct) and control cells were injected subcutaneously into nude mice to establish a xenograft tumor model. Bclaf1 repression significantly reduced the growth of tumor xenografts in nude mice (Fig. 2d). These tumor cells experienced hypoxic conditions as indicated by the high levels of HIF-1α (Fig. 2e). These results indicate that Bclaf1 contributes to tumor growth under hypoxia.

It is believed that the ability of tumor cells to survive and to adapt to hypoxic microenvironments can be mostly attributed to the stimulation of angiogenesis [29]. Among the multiple angiogenic factors, the secreted vascular endothelial growth factor (VEGF) is essential for the initiation and overall regulation of vascular growth and patterning [30]. Therefore, we visualized VEGFA and CD31 levels of tumor xenografts in nude mice by immunohistochemistry (IHC) staining; CD31 levels are indicative of microvessel density (MVD) [31]. Strikingly, depletion of Bclaf1 considerably suppressed the levels of VEGFA and CD31 (Fig. 2e), suggesting that Bclaf1 plays a role in HCC-induced angiogenesis. To investigate the relevance of Bclaf1 in angiogenesis, we next employed an in-vitro-angiogenesis model. First, we determined the extracellular concentration of VEGFA and found that it decreased after Bclaf1 depletion in hypoxia as compared to the control group grown under hypoxic conditions (left panel, Fig. 2f). In contrast, the VEGFA secretion of HCC cells was significantly increased upon BCLAF1 over-expression (Fig. 2f). Then, Human umbilical vein endothelial cells (HUVECs) were seeded on Matrigel and they migrated to establish capillary-like structures with a lumen. Compared to the control group supplied with culture supernatant of control Huh7 cells, HUVECs supplied with the culture supernatant of the same number of Bclaf1-knockdown Huh7 cells formed less-robust capillaries and cord-like structures. Consistently, the culture supernatant of the same number of BCLAF1 overexpressing Huh7 cells significantly increased the tube-formation capacity of HUVECs quantified as branching length (Fig. 2g). Similar results were observed when the culture supernatants of HepG2 cells were used. Therefore, we hypothesized that Bclaf1 is able to induce VEGFA expression to promote tumor-associated angiogenesis and consequently tumor growth. Furthermore, we assessed the sprouting of vessels from mouse aortic rings. Consistent with the tube-formation assay, treatment with medium supernatant from Bclaf1-knockdown cells resulted in less vessel sprouting at mouse aortic rings (Fig. 2h). Collectively, these results support the hypothesis that Bclaf1 is a positive regulator of angiogenesis.

### Expression of Bclaf1 correlates with HIF1A expression in the HCC

**Fig. 3:**
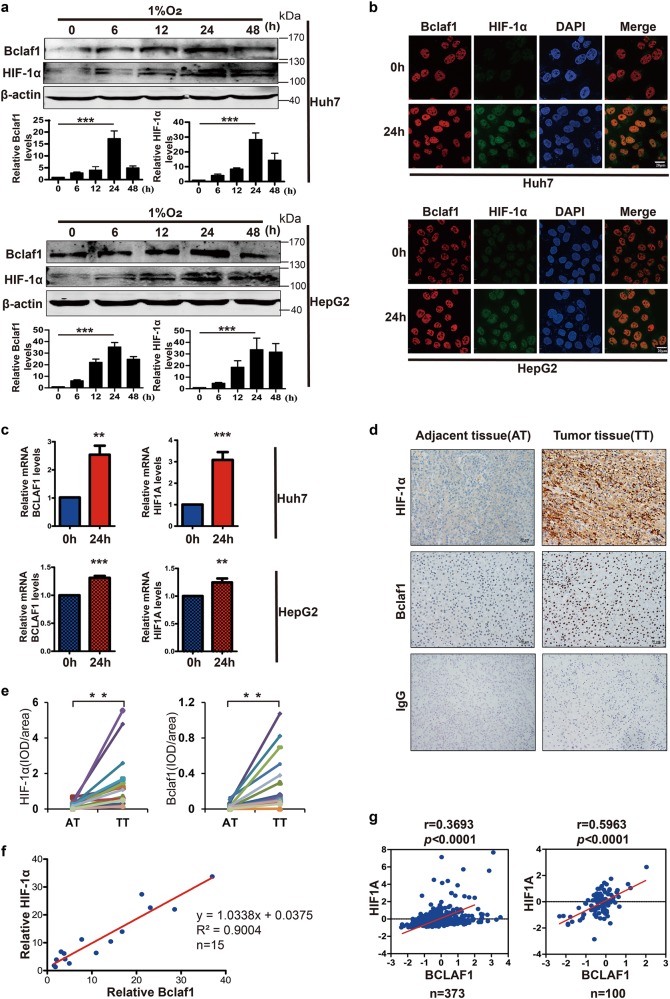
Elevated HIF-1α positively correlates with Bclaf1 in hepatocarcinoma. **a** Western blot analysis of Huh7 and HepG2 cells cultured in 1% O_2_ for 0, 6, 12, 24, 48 h. Gel-Pro software was utilized to measure the relative intensity of Bclaf1 and HIF-1α. **b**, **c** Immunofluorescence staining and qPCR were employed to observe the Bclaf1 and HIF-1α protein amounts and respective mRNA levels after Huh7 and HepG2 cells were cultured in 1% O_2_ for 24 h. **d** Immunohistochemistry of Bclaf1 and HIF-1α in the tumor and adjacent tissues of hepatocarcinoma patients. **e** Statistical analysis of normalized levels of Bclaf1 and HIF-1α (integrated optical density [IOD] against immunoglobulin G [IgG]) in the tumor and adjacent tissues of hepatocarcinoma patients. **f** Correlation between fold increase of Bclaf1 and HIF-1α relative to adjacent tissues in the above-shown tumor subjects. **g** Correlation between BCLAF1 and HIF1A mRNA levels in the publicly available database by cBioportal and the publicly available microarray database (GSE62043), respectively. Scale, log2 median-centered value of gene expression. Pearson coefficient tests were conducted to calculate statistical significance. **a**–**c** Data represent mean ± SD of three independent experiments. *p < 0.05, **p < 0.01, ***p < 0.001 vs. control

To test whether the Bclaf1 levels correlate with HIF-1α levels under hypoxia, we cultured HepG2 and Huh7 cells for different time intervals (0, 6, 12, 24, 48 h) in 1% O_2_. The levels of Bclaf1 and HIF-1α increased significantly during the first 24 h hypoxia treatment and were again decreased by 48 h (Fig. 3a). Therefore, we chose a hypoxia exposure time of 24 h for the following experiments. Consistently, immunofluorescence staining by confocal microscopy revealed more intense staining of Bclaf1 and HIF-1α after hypoxia treatment for 24 h (Fig. 3b). Accordingly, the mRNA levels of BCLAF1 and HIF1A were also higher in HCC cells incubated for 24 h under hypoxia than in control cells grown under normoxic conditions (Fig. 3c). These findings reveal that increased Bclaf1 parallels HIF-1α levels under hypoxia in HCC cells.

We next studied whether the levels of Bclaf1 and HIF-1α correlate in human HCC tissue samples. To differentiate between the neoplastic and normal regions, tissue sections were stained with hematoxylin–eosin and validated independently by two pathologists. HIF-1α and Bclaf1 levels were significantly higher in tumor tissues than in adjacent normal tissues (Fig. 3d, e), and the levels of Bclaf1 and HIF-1α were positively correlated (Fig. 3f; *r* = 0.9004, *p* < 0.01). In addition, data from publicly available databases revealed a positive correlation between the mRNA level of BCLAF1 and HIF1A in 373 HCC patients (cBioportal [32, 33], Fig. 1g) and in 100 HCC patients (microarray database GSE62043 [34]; Fig. 1g), suggesting that BCLAF1 is highly expressed in tumor hypoxic microenvironment and correlated with HIF1A.

### Bclaf1 regulates HIF1A transcription under hypoxia

**Fig. 4:**
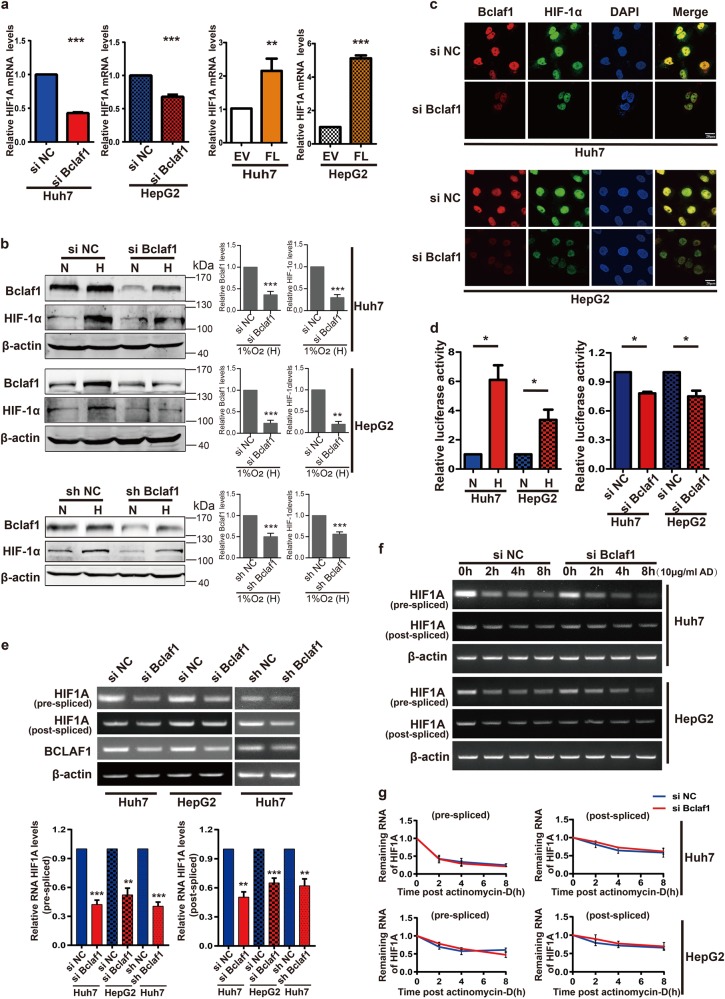
Bclaf1 regulates HIF-1α transcription in hypoxia. Huh7 and HepG2 cells were transfected with indicated plasmids and 24 h post-transfection cultured in hypoxia (1% O_2_) for additional 24 h. **a**, **e** HIF1A mRNA levels were assessed by RT-qPCR (**a**) and RT-PCR (**e**). **b**, **c** Protein levels of Bclaf1 and HIF-1α were detected by Western blotting (**b**) and immunofluorescence staining (**c**). **d** After transfecting HCC cells with a luciferase reporter of the HIF-1 promoter response elements (HRE) or co-transfecting with siRNA, cells were cultured under normoxia (N) or hypoxia (H; 1% O_2_) for 24 h. Relative luciferase activity of the reporters was determined and normalized to the control group. **f**, **g** HCC cells were transfected with siNC or siBCLAF1 for 24 h, then treated with 10 μg/ml actinomycin D prior to RNA extraction at the time points indicated. HIF1A mRNA levels were analyzed by RT-PCR and Gel-Pro software. All data represent mean ± SD of three independent experiments. *p < 0.05, **p < 0.01, ***p < 0.001 vs. control

Since the above-presented data indicate a correlation between Bclaf1 and HIF-1α levels, we wondered whether Bclaf1 directly regulates HIF1A expression under hypoxia. Consistent with this hypothesis, the abundance of HIF1A mRNA significantly decreased upon BCLAF1 down-regulation and dramatically increased upon BCLAF1 overexpression (Fig. 4a), suggesting that Bclaf1 acted on HIF1A at the mRNA level. In parallel with the mRNA changes, HIF-1α protein levels changed consistently: down-regulation of Bclaf1 levels by transient siRNA transfection or stable shRNA transfection reduced hypoxia-induced HIF-1α protein levels (Fig. 4b). Similarly, immunofluorescence staining also confirmed that Bclaf1 knockdown decreased HIF-1α levels under hypoxia (Fig. 4c). These data imply that the expression of the HIF1A gene is modulated by Bclaf1. To further evaluate the role of Bclaf1 in the expression of HIF-1α target genes, we used a luciferase reporter plasmid driven by the human HIF-1 response elements (HRE) to detect the transcriptional activity of HIF-1. Consistent with a previous study [35], we detected a significant increase in HRE dependent luciferase activity under hypoxia. When cells were transiently cotransfected with this reporter plasmids along with BCLAF1 siRNA, a clear decrease in reporter activity was observed (Fig. 4d). Taken together, these findings support the hypothesis that Bclaf1 regulates HIF1A transcription under hypoxia.

Bclaf1 was shown to be involved in pre-mRNA biogenesis and processing events, such as pre-mRNA splicing, mRNA localization and stabilization [22, 36]. Hence, we wondered which step in HIF1A RNA processing is Bclaf1-dependent. Using primer pairs specifically designed to amplify the pre-splicing (pre-mRNA) and the post-splicing (mature) mRNA of HIF1A, we found that depletion of Bclaf1 could decrease the levels of HIF1A pre-mRNA and HIF1A mature mRNA in HepG2 and Huh7 cells (Fig. 4e). HIF-1α protein levels changed with the same trend (Fig. 4b). These findings suggested that the Bclaf1-induced effect on HIF1A expression is primarily transcriptional. To check the effect of Bclaf1 on HIF1A mRNA splicing and stability, we treated the cells with the RNA polymerase II inhibitor actinomycin D (ActD) for different time intervals (0, 2, 4, 8 h) with or without Bclaf1 knockdown. Though both, pre-mRNA and mature mRNA of HIF1A decreased after the inhibition of transcription, the level of pre-mRNA decreased more significantly than the mature mRNA. However, the decrease of both mRNA species was similar in cells depleted for Bclaf1 by siRNA as in cells with wild type levels of Bclaf1, indicating that Bclaf1 neither affects splicing of HIF1A pre-mRNA nor stability of mature HIF1A mRNA (Fig. 4f, g). Collectively, these data demonstrate that under hypoxia Bclaf1 regulates HIF1A expression solely at the transcriptional level.

### HIF1A transcription is controlled by Bclaf1 through its bZIP DNA-binding domain

**Fig. 5:**
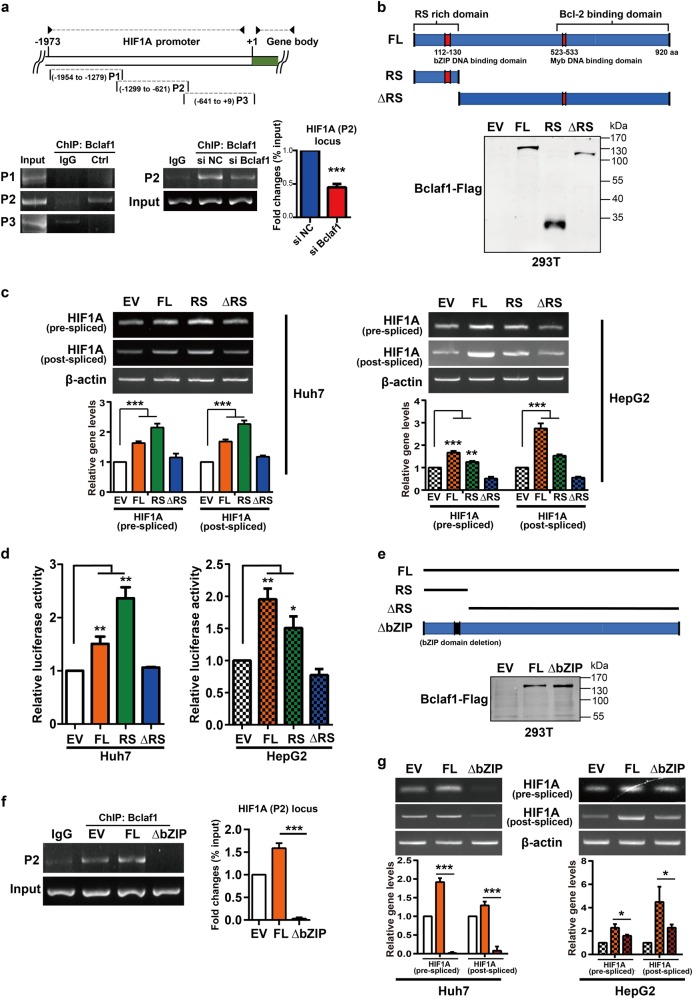
Bclaf1 upregulates HIF1A transcription through its bZIP DNA-binding domain. Huh7 and HepG2 cells were transfected with the indicated plasmids and 24 h post-transfection cultured in hypoxia (1% O_2_) for 24 h. **a** The schematic representation of HIF1A promoter with the indicated regions (P1 to P3) for PCR analysis. The position is relative to the start of the first exon. ChIP analysis of HIF1A promoter of siNC (control) or siBCLAF1 transfected Huh7 cells exposed to hypoxia for 24 h. Immunoprecipitation was performed using anti-Bclaf1 and IgG antibodies followed by PCR analysis. **b**, **e** A schematic representation of the Bclaf1 protein. Bclaf1 domain levels in 293T cells transfected with the indicated plasmids analyzed by immunoblotting. **c**, **f** The HIF1A mRNA levels were assessed by RT-PCR. **d** Relative luciferase activity of the HIF-1 reporter was examined after hypoxia exposure. **g** ChIP analysis of HIF1A promoter in Huh7 cells exposed to hypoxia for 24 h after transfection with plasmids encoding the indicated domains. Immunoprecipitation and PCR are performed as described in (**a**). Data represent mean ± SD of three independent experiments. *p < 0.05, **p < 0.01, ***p < 0.001

The Bclaf1 protein contains a basic zipper (bZIP) homologous and a Myb homologous DNA-binding motif, and can bind to DNA and function as a regulator of transcription [20]. First, we performed a chromatin immune precipitation (ChIP) assay to examine whether Bclaf1 binds to the promoter region of the HIF1A gene. We designed three sets of primers that span the sequence from +9 to −1954 bp (Fig. 5a, +1 indicates the first bp of exon 1) of the HIF1A promoter. From the Bclaf1 ChIP samples, only the P2 region could be amplified, indicating that Bclaf1 specifically binds to the P2 region of the HIF1A promoter (left panel, Fig. 5a). Moreover, Bclaf1 depletion significantly decreased the binding of Bclaf1 to the HIF1A promoter (right panel, Fig. 5a, supplemental Fig. S1e), consistent with our previous results (Fig. 4).

To further elucidate which domain of Bclaf1 is essential for HIF1A transcription, we prepared expression vectors encoding FLAG fusion constructs of different domains of Bclaf1, encompassing the sequences for full-length Bclaf1 (FL), a Bcl-2 binding domain deleted Bclaf1 mutant (RS), an RS domain deleted mutant (∆RS), and the empty vector (EV) as control (Fig. 5b, supplemental Fig. S1a, b). HCC cells were transfected with the different vectors and cultured in hypoxia for 24 h. Transfection of Huh7 cells with the RS domain-encoding vector increased pre-mRNA and mature HIF1A mRNA abundance even more than transfection with the vector encoding full-length BCLAF1. In HepG2 cells, full-length Bclaf1 caused a more prominent increase in HIF1A mRNA than the RS domain construct. In contrast, upon transfection with the vector encoding the RS domain deleted variant (∆RS) no increase in HIF1A mRNA as compared to the empty vector control was observed (Fig. 5c, supplemental Fig. S1c). Similar changes were observed in the transcriptional activity of HIF-1 by cotransfecting cells with the HRE luciferase reporter plasmid (Fig. 5d). Taken together, these data demonstrate that HIF1A transcription is controlled by the RS domain of Bclaf1 under hypoxia.

To further confirm whether the Bclaf1-mediated regulation of HIF1A transcription is associated with the basic zipper (bZIP) homologous DNA-binding motif of the Bclaf1 RS-domain, we designed vectors encoding FLAG-Bclaf1 fusion constructs of a bZIP motif deleted variant (∆bZIP) (Fig. 5e). The presence of the bZIP motif was essential for Bclaf1 to bind to the P2 promoter region of the HIF1A gene (Fig. 5f) and to mediate the effect on HIF1A expression (Fig. 5g, supplemental Fig. S1d). All of these data show that Bclaf1 regulates HIF1A transcription via the bZIP DNA-binding domain.

### Bclaf1 is required for HIF-1α-mediated expression of angiogenic genes

**Fig. 6:**
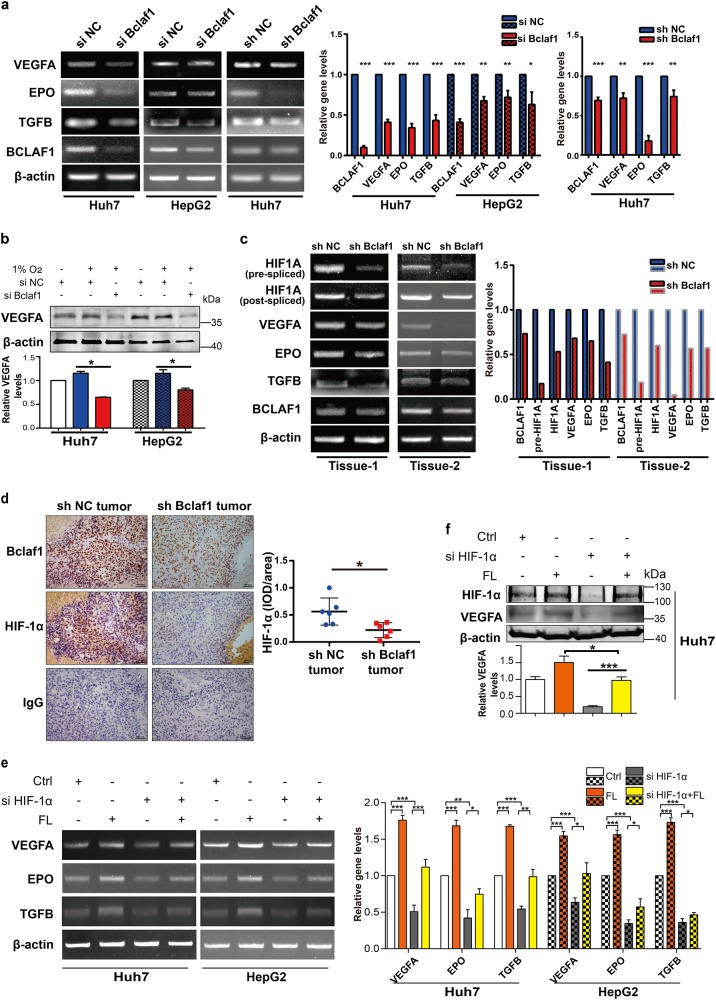
Bclaf1 upregulates the downstream angiogenesis targets of HIF-1α. **a** Huh7 and HepG2 cells were transfected with siNC/shNC (negative control) or siBCLAF1/shBCLAF1, cultured for 24 h, and subsequently treated in hypoxia (1% O_2_) for 24 h. The mRNA levels of VEGF, EPO, TGFB, and BCLAF1 were detected by RT-PCR. **b** HCC cells were transfected with siNC or siBCLAF1. 24 h post-transfection the cells were untreated or treated in hypoxia for 24 h followed by SDS-PAGE and western blot analysis. Color code as in (**a**). **c** shNC or shBCLAF1 stably transfected Huh7 cells were injected into the subcutaneous area of nude mice (*n* = 6). The mRNA levels of HIF1A, VEGFA, EPO, TGFB, and BCLAF1 from two representative xenograft tumor tissues were detected by RT-PCR. **d** Immunohistochemistry (200×) of HIF-1α and Bclaf1 in the xenograft tumor tissues. Statistical analysis of normalized levels of HIF-1α (integrated optical density [IOD] against immunoglobulin G [IgG]) was analyzed with Image pro 6.0. **e**, **f** The mRNA and protein levels were detected by RT-PCR and western blot after transfection with indicated siRNAs or expression plasmids. Data represent mean ± SD of three independent experiments. *p < 0.05, **p < 0.01, ***p < 0.001

It is well known that activated HIF-1 plays a crucial role in the adaptive responses of tumor cells to changes in oxygen partial pressure through activation of transcription of over 100 downstream genes, including the genes encoding VEGF, transforming growth factor-β (TGF-β), and erythropoietin (EPO) that are involved in angiogenesis and cell proliferation [25]. To further investigate the effect of Bclaf1 on HIF-1α downstream angiogenesis genes, we down-regulated Bclaf1 by transient siRNA or stable shRNA transfection of HCC cells. We detected a significant decrease in the mRNA levels of VEGFA, EPO, and TGFB (Fig. 6a, supplemental Fig. S2a), in parallel with a dramatically reduced concentration of the VEGFA protein (Fig. 6b). Consistent with the cell model, the in vivo amount of VEGFA, EPO, TGFB mRNA were also much lower in xenografts derived from shBcaf1 cells than in control xenografts (Fig. 6c). Also, in tumor tissues, depletion of Bclaf1 considerably suppressed the expression of HIF1A (Fig. 6d), explaining the decrease of VEGFA protein levels (Fig. 2). These data strongly suggest that Bclaf1 suppresses downstream angiogenic genes of HIF-1α in vivo and in vitro.

To verify that Bclaf1 overexpression-induced up-regulation of VEGFA, TGFβ, and EPO is mediated through HIF-1α, we co-transfected a vector encoding full-length Bclaf1 (FL) with HIF1A-specific siRNA. When HIF-1α levels were increased by Bclaf1 overexpression, mRNA levels of down-stream angiogenesis genes were up-regulated, an effect that was inhibited by HIF-1α knockdown (Fig. 6e, supplemental Fig. S2d). VEGFA protein levels followed this trend of the VEGFA mRNA (Fig. 6f). Our data demonstrate that Bclaf1 affects angiogenic gene expression directly through HIF-1α. Taken together, these in vitro and in vivo results demonstrate that HIF-1α and its downstream angiogenesis-related genes are important targets of Bclaf1 that play a crucial role in HCC-associated hypervascularization.

## Discussion

**Fig. 7:**
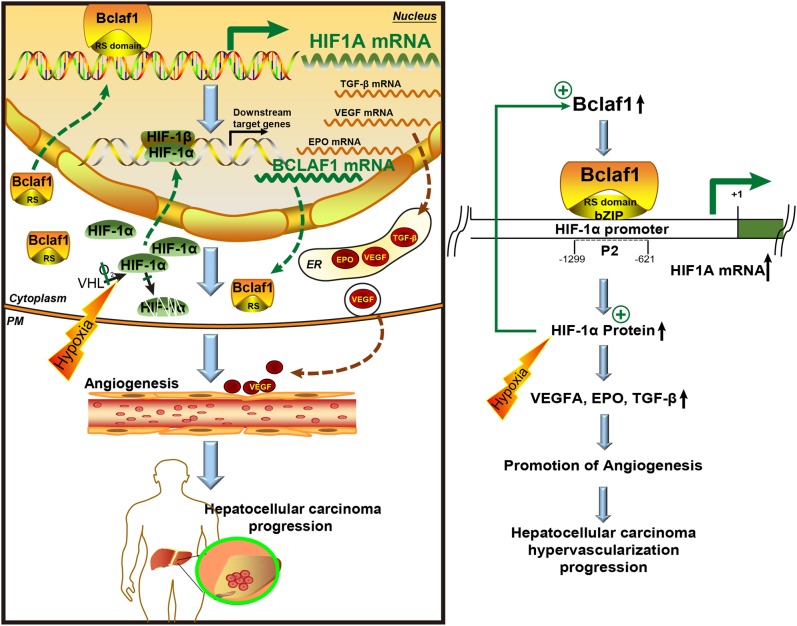
Model for the Bclaf1-HIF-1α control circuit. Hypoxic conditions reduce the hydroxylation and VHL-dependent degradation of HIF-1α. Low constitutive expression of HIF1A leads to slowly increasing HIF-1α levels that drive transcription of BCLAF1, inducing increasing levels of Bclaf1. Bclaf1 promotes transcription of HIF1A by directly binding to the HIF1A promoter, and consequently leads to the increase of HIF-1α protein. This positive feed-back loop leads to rapid increase of both, HIF-1α and Bclaf1. Upregulated HIF-1α protein induces the expression of HIF-1α downstream targets genes, producing among others VEGF, EPO, TGF-β that are essential for angiogenesis and tumor growth, consequently promoting HCC neovascularization and progression

In this study, we demonstrate that Bclaf1 promotes HIF-1α-mediated angiogenesis by binding to the promoter of HIF1A gene and directly activating transcription under hypoxic conditions (Fig. 7). These in vitro data explain why levels of Bclaf1, HIF-1α, and HIF-1α targets positively correlate with each other in clinical HCC samples, and also correlated with tumor weight in a xenograft HCC tumor model. Thus, we propose that Bclaf1 fosters liver cancer growth and progression through transcriptional regulation of HIF-1α and its downstream angiogenic target genes, ultimately providing potential strategies and biomarkers for optimally developing novel anti-angiogenic therapies of HCC and potentially other solid tumors.

Hypoxia is a common condition in solid tumors and is particularly frequent in HCC due to its rapid growth [4]. Consistent with the notion that hypoxia is a major challenge for growth, malignity, and metastasis of solid tumors, a large number of genes including VEGF, TGFB, and EPO, involved in different steps of angiogenesis are up-regulated in clinical specimens [6, 26, 37], suggesting that only those solid tumors become clinically apparent that were able to overcome the hypoxia hurdle. Our study now links Bclaf1 to this crucial transition in HCC.

The canonical mechanism of HIF-1α accumulation is controlled by VHL (von Hippel–Lindau) E3 ubiquitin ligase complex, through oxygen-dependent proline hydroxylation. Under normoxia, HIF-1α protein is synthesized constitutively, but degraded rapidly by the VHL-mediated ubiquitin-proteasome system [4, 38]. In a hypoxic microenvironment, such as in solid tumors, HIF-1α escapes proline hydroxylation, VHL binding and degradation, hence causes the transcription of genes that contain the hypoxia response element to drive expression of many genes involved in glycolysis, angiogenesis, cell survival, and cancer progression [4]. Whereas the regulation of HIF-1α protein is well documented, little is known about the regulation and turnover of HIF1A mRNA. Our RNA-seq results of HCC cells depleted for Blcaf1 indicate that Bclaf1 depletion significantly down-regulates HIF1A mRNA level, as compared to VHL mRNA level (Fig. 1d, left panel). Consistent with these results, siBCLAF1 led to decreased HIF-1α levels, without obvious effects on degradational and translational pathways (supplemental Fig. S3a, b).

Bclaf1 was originally reported with a role in either promotion of apoptosis or cell cycle arrest [15, 39]. Subsequently, it was identified as a tumor suppressor through inhibition of cell proliferation by microRNAs in bladder cancer [40] and by interaction with H2AX in lung adenocarcinoma [41]. However, recent studies unearthed the dark side of Bclaf1, revealing its oncogenic features in acute myeloid leukemia [42] and colon cancer cells [24]. Bclaf1 also enhances proliferation of endothelial cells, in the context of delivery of microRNA miR-143-3p via platelet-derived microparticles in a hypertension model [43]. These diverse observations suggest that Bclaf1 is involved in a wide range of biological processes. However, there were no indications to suggest a relationship between Bclaf1 and hypoxia-induced angiogenesis that could facilitate the growth of solid tumors.

Several studies assigned a regulatory role for Bclaf1 in transcription [40, 41], such as Bclaf1 is an activator of transcription through core promoter elements [41] and through direct interaction of the Myb motif of Bclaf1 with the bZIP motif of C/EBPβ [20]. Here, we found that Bclaf1 supports HCC-associated angiogenesis by driving transcription of HIF1A. Analyzing the sequence of HIF1A promoter revealed that there are also recognition sites for C/EBPβ and the activation of HIF1A transcription could in principle also involve C/EBPβ. However, for the induction of HIF1A transcription, the RS domain of Bclaf1 was essential, whereas the RS domain was not necessary for interaction with C/EBPβ [20]. On the other side, Myb motif of Bclaf1 was essential for the interaction with C/EBPβ, whereas it was dispensable for the induction of HIF1A transcription (Fig. 5). We therefore conclude that Bclaf1 induces HIF1A transcription by a distinct mechanism.

Other recent studies also suggested a role for Bclaf1 in pre-mRNA splicing and processing alterations and mRNA stabilization [37]. Recently, we found that Bclaf1 supports HCC growth by regulating the oncogenic driver c-MYC at the post-transcriptional level by preventing degradation of the mature c-MYC mRNA [44]. In addition, earlier studies declared that c-Myc post-transcriptionally regulates HIF1α expression under hypoxia [45]. However, there are two arguments that the here-observed influence of Bclaf1 on HIF1A transcription is not mediated via c-Myc. First, c-Myc has no significant effect on HIF1A transcription (supplemental Fig. S4a, b). Second, we demonstrate that Bclaf1 directly binds to the promoter of the HIF1A gene through its bZIP DNA binding motif.

Since Bclaf1 can also act at the post-transcriptional or even the post-splicing stage, it would be possible that Bclaf1 influences HIF-1α expression additionally at these stages of gene expression. However, we do not favor such a hypothesis since the ActD-induced decrease of the HIF1A pre-mRNA was more prominent than the decay of the mature HIF1A mRNA (Fig. 4f, g). If Bclaf1 had a positive effect on splicing of HIF1A pre-mRNA or on the stability of mature HIF1A mRNA, then the amount of mature mRNA should decrease faster than the amount of the pre-mRNA upon Bclaf1 knockdown. This is not what we observed. Also, levels of HIF-1A mature mRNA should decrease faster upon Bclaf1 knockdown than in control cells with wild type levels of Bclaf1 contrary to our observations (Fig. 4g).

Interestingly, when analyzing the domains of Bclaf1 that are responsible for driving the transcription of HIF1A (Fig. 5c) and the HRE luciferase reporter construct (Fig. 5d), we noticed that ectopic expression of a gene encoding only the RS domain of Bclaf1 was more efficient in Huh7 cells than ectopic expression of the full-length BCLAF1 gene, indicating not only that the RS domain is necessary and sufficient for the Bclaf1 effect but also that other domains of Bclaf1 seem to act inhibitory on transcription activation, potentially by interacting with transcription factors that downregulate transcription or by displacing transcription co-activators. Furthermore, ectopic expression of a BCLAF1 construct with deleted coding sequence for the RS domain, producing a Bclaf1 variant that does not bind to the HIF1A promoter, resulted in HIF1A pre-mRNA and mature mRNA levels and an HRE luciferase reporter activity that were similar to the control cells transfected with the empty vector, indicating that Bclaf1 domains outside the RS domain only affect HIF1A expression in Huh7 cells when the Bclaf1 is bound to the HIF1A promoter. In contrast, in HepG2 cells, ectopic expression of the RS domain encoding gene led to a significantly smaller increase of HIF1A pre-RNA and mature RNA and in HRE luciferase reporter activity than ectopic expression of the full-length BCLAF1 gene, suggesting that other domains of Bclaf1 contribute to the transcription activation activity of Bclaf1, potentially by recruiting transcriptional co-activators present in HepG2 cells but not in Huh7 cells to the HIF1A promoter. Moreover, ectopic expression of a BCLAF1 construct with deleted coding sequence for the RS domain led to slightly lower concentrations of HIF1A pre-mRNA and mature mRNA than the levels in the control cells, suggesting that the overexpressed Bclaf1 without the RS domain had a slight dominant negative effect in the presence of the endogenous Bclaf1. Consistent with these observations, deletion of the bZIP motif in Bclaf1 almost completely abrogated HIF1A transcription in Huh7 cells but still had a positive effect on HIF1A transcription in HepG2 cells (Fig. 5f). These differences between Huh7 and HepG2 cells are intriguing and will need further investigation in future studies to identify additional transcription coactivators or factors that limit the up-regulation of HIF1A transcription. Such differences might be important for malignity of the cancer cells and potentially for the development of HIF1A transcription inhibiting drugs.

Intriguingly, we observed that Bclaf1 protein levels are significantly increased in hypoxic conditions (Fig. 2a) and that HIF1A silencing reduces Bclaf1 protein levels in hypoxia (supplemental Fig. S5a, b). Since we also indicate that Bclaf1 drives transcription of HIF1A, HIF-1α, and Bclaf1 form a positive feed-back loop, i.e., increasing levels of Bclaf1 protein stimulate transcription of the HIF-1A gene and in turn, increasing levels of HIF-1α protein induce in rising levels of Bclaf1. Such positive feedback loops are extremely sensitive and allow for a rapid, self-amplifying response in a switch-like fashion but also raises the question of how such a system is kicked off in the first place and how it can be limited or shut off. As mentioned above, HIF-1α is not only regulated at the transcriptional level but also at the protein level by posttranslational oxidation leading to rapid degradation by the ubiquitin-proteasome pathway. Hypoxic conditions slow down HIF-1α hydroxylation and subsequent degradation and the low level of constitutive transcription of the HIF1A gene will lead to a moderate accumulation of HIF-1α. Since HIF-1α drives transcription of BCLAF1 and Bclaf1 drives transcription of HIF1A in a positive feed-back loop, even a small initial increase in HIF-1α will be rapidly amplified leading to a substantial increase of both HIF-1α and Bclaf1. In this study, we show that increased Bclaf1 promotes HIF-1α-mediated angiogenesis. And the assumption could be that the increased angiogensis facilitates oxygen transport, leading to increasing O_2_ levels. Then the increasing O_2_ partial pressure will lead to hydroxylation of HIF-1α with subsequent ubiquitination and degradation, breaking the positive feedback loop, resulting in reducing Bclaf1 expressionand concomitant reducing HIF1A expression. The HIF-1α-mediated increase in Bclaf1 levels also possibly solves another conundrum. One puzzling observation is that hypoxia spurs the growth of HCC cells. A priori we would expect that reduced availability of O_2_ should decrease the O_2_-dependent metabolism and ATP levels in the cell and thus should limit cell division. However, as we previously showed, increased Bclaf1 levels stabilize mature c-MYC mRNA with subsequent increased c-Myc protein levels [44]. In turn, c-MYC is known to activate various mitogenic signals.

In summary, our study identified an important novel oncogenic function for Bclaf1, which is at the same time upstream and downstream of HIF-1α, linked in a positive feedback loop (Fig. 7). Bclaf1 enhances transcription of HIF1A during hypoxia and through the HIF-1α transcription factor a large number of downstream genes, many of which are involved in angiogenesis. Since hypoxia is a critical condition limiting the growth of solid tumors, this pathway plays a vital role in HCC progression. Since Bclaf1 also stabilizes the mRNA encoding the tumor driver oncogene c-Myc [44], Bclaf1 promotes HCC at two different levels, proliferation and growth supporting vascularization in a synergistic way. Our study therefore supports the proposal that Bclaf1 could be a target for anti-proliferative and anti-angiogenic therapy of HCC.

## Materials and methods

The following methods are detailed in Supplementary Information, including the source of human tissue, mouse xenotransplantation experiments, cell culture and reagents, cell proliferation assay, western blotting, immunofluorescence, PCR, transfection of siRNA and plasmids, IHC, Dual-Luciferase assay, chromatin immunoprecipitation (ChIP), ELISA, tube formation assay, aortic ring assay, RNA-seq, and data analysis.

## Electronic supplementary material

Supplementary materials methods figure legends

Fig.S1

Fig.S2

Fig.S3

Fig.S4

Fig.S5

Tab.S1
