# Supplementary material for: Bclaf1 promotes angiogenesis by regulating HIF-1α transcription in hepatocellular carcinoma
Source: Oncogene. 2018 Oct 26;38(11):1845–59. doi: 10.1038/s41388-018-0552-1 (PMC6462866; doi:10.1038/s41388-018-0552-1)
Supplement: Supplementary file 1 — Supplementary materials methods figure legends [file 41388_2018_552_MOESM1_ESM.docx]

# Supplementary materials and methods

**Human tissue**

This study was approved by the Institutional Review Board of the first affiliated hospital of Guangdong pharmaceutical university (Guangzhou, China), all patients provided written informed consent for the use of surgical samples. Fresh HCC tissue samples and corresponding adjacent noncancerous tissues were obtained from 15 patients who were diagnosed with HCC in the first affiliated hospital of Guangdong pharmaceutical university (Guangzhou, China). These samples were collected from patients during surgery. None of the patients had been preprocessed with radiotherapy or chemotherapy before undergoing a hepatectomy. The details of human tissues are described in the supplementary Information table s1.

**Mouse xenotransplantation experiments**

BALB/c male nude mice (Four weeks old, Animal Center of Southern Medical University) were housed three mice per cage under specific pathogen-free condition. Cells (1× 10^7^ in 200 µl serum-free media) were subcutaneously injected into the flanks of nude mice. There were 6 animals in each experimental group. The experimental groups were randomly assigned for different treatments. We will ensure >4 mice per group to get 90% power at *p* < 0.05 using ANOVA. Tumors were further processed for Immunohistochemistry. This study was approved by the IACUC (Institutional Animal Care and Use Committee) of Nanfang Hospital affiliated with the Southern Medical University (Approval code L2015069).

**Cell culture and reagents**

Human liver cancer cell line HepG2 and Huh7 cells were purchased from the Type Culture Collection of the Chinese Academy of Sciences, Shanghai, China. The cells were confirmed free from mycoplasma contamination with [MycAwayTM-Color One-Step Mycoplasma Detection Kit](http://www.yeasen.com/Mycoplasma/1645.htm) (Yeasen, Shanghai, China). Then the cells were cultured in Dulbecco’s modified Eagle’s medium (Gibco, USA) with 10 % fetal bovine serum (Gibco, USA). Normoxia or hypoxia conditions were maintained at 37 °C in the incubator with 20 % O_2_ and 5 % CO_2_ or 94 % N_2_, 5 % CO_2_, and 1 % O_2_. ActinomycinD was obtained from Jetway (Guangzhou, China). MG-132 and CHX were obtained from SelleckChem (Houston, TX, USA).

**Cell proliferation assay**

Cell proliferation was measured with a cell counting kit-8 (CCK8) assay (Dojindo, Tabaru, Mashikimachi, Japan) and crystal violet staining assay. CCK8: 2 × 10^3^ cells per well of Huh7 cells were seeded into 96-well plates in 100 μL medium and incubated for 24 h, after treatments, fresh medium with 10% CCK-8 was substituted for the previous. Absorbance of 450 nm was measure after a 2-h incubation at 37°C. Three replicate wells were included in each group. Crystal violet: 1 × 10^5^ cells per well of Huh7 cells were seeded into 6-well plates and incubated for 24 h, after treatments, cells were stained with 0.5% crystal violet solution in 20% methanol after washed with PBS. Pictures (200×) of the wells were taken using a wide-field microscopy (Olympus, Tokyo, Japan) and the number of cells were scored using the Image-J software.

**Western blotting**

Lysis buffer containing protease inhibitor (Pierce, MA, USA), 20 mM PIPES (pH=7.0), 100 mM NaCl, 2 mM Na_3_VO_4_, 20 mM Na_2_MoO_4_, 1 mM MgCl_2_, 30 mM NaF, 1% Triton X-100 was used to lyse the cells as before([1](#_ENREF_1)). Protein concentrations of the cell lysates were determined by the BCA method (Bio-Rad, CA, USA). Each sample (40 μg) was separated by 10 % SDS-PAGE and transferred onto a PVDF membrane. Primary antibodies: β-actin (Ray Antibody Biotech, Beijing, China; RM2001), Bclaf1 (Santa Cruz Biotechnology, CA, USA; sc-135845 and Sigma Aldrich, MO, USA; HPA027770), HIF-1α (Abcam, MA, USA; [ab16066)](https://www.abcam.cn/hif-1-alpha-antibody-mgc3-ab16066.html)), VEGFA (Sigma Aldrich, MO, USA; ab46154). Secondary antibodies (IRDye 680/800) were from LI-COR Biosciences. Blots were scanned by Li-COR Odyssey infrared imaging system and analyzed by Gel-pro analyzer 4([1](#_ENREF_1)).

**Immunofluorescence**

Cells were filled with 4% paraformaldehyde for 10 minutes at room temperature, then washed with cold PBS. Permeation was performed by incubating pretreated frozen methanol at -20°C for 15 min. Cells were incubated with [primary](file:////C:\Program%2520Files%2520(x86)\Youdao\Dict\7.0.1.0227\resultui\dict\%3fkeyword=primary) [antibodies](file:////C:\Program%2520Files%2520(x86)\Youdao\Dict\7.0.1.0227\resultui\dict\%3fkeyword=antibodies) (1:100) at 4°C overnight. Then cells washed with cold PBS and were incubated with Alexa Fluor 555 donkey anti-rabbit IgG (Life technologies, CA, USA) (1:200) or Alexa Fluor 488 donkey anti-mouse IgG (Life technologies, CA, USA) (1:200) at room temperature for 2 h. After the nuclei were stained with DAPI, images were captured using an Olympus FV1000 Confocal Laser Scanning Microscopy (Tokyo, Japan).

**PCR**

Total cell RNA was isolated by RNAiso (Takara, Tokyo, Japan). 1000 ng of total RNA was used to reverse transcription with Prime Scrip RT reagent Kit (Takara). Real-time quantitative PCR and PCR were performed using SYBR Premix Ex Taq and Premix Taq (Takara) respectively with the indicated primer (Supplementary table s2). The amplification curves and melting peaks of primer pairs are shown in Supplementary table s3.

**Transfection of siRNA and plasmids**

Cells were transfected with the indicated siRNA oligos (Gene Pharma, Shanghai, China) or plasmid with Lipofectamine 3000 (Invitrogen, CA, USA) for 48h (including hypoxic exposed time), all steps were performed according to the manufacturer’s instructions. The shBCLAF1 plasmid containing cells were selected in the presence of G418 (800 µg/mL) for 2–3 weeks. The successfully transfected/knockdown cells were confirmed by Western blotting. The sequences contained siBclaf1 (F5′- GCAGAGGGCGCUUUAACUUTT-3′, R 5′- AAGUUAAAGCGCCCUCUGCTT-3′), siHIF-1α (F5′-GGCCGCUCAAUUUAUGAAUTT-3′, R5′-AUUCAUAAAUUGAGCGGCCTT-3′), shBCLAF1 (F5′-GGTGGTTATAGACCTGTCTGG-3′, R5′-CCAGACAGGTCTATAACCACC-3′); sic-Myc (F5′-CCACACAUCAGCACAACUATT-3′, R 5′-UAGUUGUGCUGAUGUGUGGTT-3′). The Bclaf1 plasmids of over express full length and its different domain were kindly provided by Dr. Tang (College of Veterinary Medicine, China Agricultural University). Then, we designed vectors expressing FLAG fusion constructs encoding a bZIP domain (112-130aa) deleted Bclaf1 mutant (∆bZIP).

**Immunohistochemistry (IHC)**

IHC was performed on paraffin sections of tissues according to the standard LSAB protocol (BOSTER, Guangzhou, China), using primary antibodies against Bclaf1 (1:100), HIF-1α (1:100), VEGFA (1:350), CD31 (ABclonal, China; A3181) (1:300). Isotype-matched IgG was used as negative controls. Statistical analysis of normalized expression of Bclaf1 and HIF-1α (integrated optical density [IOD] against immunoglobulin G [IgG]) in the tumor and adjacent tissues of hepatocarcinoma patients or xenograft tumor nude mice were analyzed by Image-Pro Plus 6.0.

**Dual-Luciferase assay**

Cells were treated as described in the result section and Dual-Luciferase assay was then performed according to the standard Promega (Madison, WI, USA) protocol. Relative luciferase activity was expressed as normalized with the control group. Plasmid vectors of the HIF-1 reporter plasmid was synthesized by Genomeditech (Beijing, China).

**Chromatin immunoprecipitation (ChIP)**

ChIP experiments were conducted using the published protocol for the fast chromatin immunoprecipitation method([2](#_ENREF_2)). Chromatin was diluted in ChIP buffer (150 mM NaCl, 50 mM Tris HCl, pH 7.5, 5 mM EDTA, 0.5% NP-40, 1.0% Triton X-100 and protease inhibitors; Roche). DNA was disrupted by sonication at 50% amplitude for 30 secs with 2 mins intervals on ice. Ultrasonic systems (Scientz JY88-Ⅱ, China) was optimized to produce DNA segments ranging from +100 to + 500 bp as determined by agarose gel electrophoresis. Bclaf1 was precipitated by incubating the cell extract with 2 µg of Bclaf1-specific antisera and overnight incubation. The complexes were captured using Protein A/G-Sepharose beads (Millipore, MA, USA). After de-crosslinking, the DNA samples were analyzed by Real-time Quantitative PCR (Roche) and electrophoresis on a 3% agarose gel.

**ELISA**

VEGF concentration in cell culture medium was determined by ELISA using the Human VEGF ELISA Kit according to the manufacturer’s instructions (Cusabio, Wuhan, China). For Huh7 cells 1 × 10^5^ cells per well and for HepG2 cells 1 × 10^3^ cells per well were seeded into 6-well plates and incubated for 24 h, after treatments, before VEGF concentrations were determined. The results of the ELISA assay were expressed as pg/mg.

**Tube formation assay**

Tube formation was assayed as previously described([3](#_ENREF_3)). In brief, 50 μL growth factor-reduced Matrigel (BD Biosciences, NY, USA) was pipetted into 96-well plates and polymerized for 30 minutes at 37°C. HUVECs incubated in F12/DMEM medium containing 1% FBS for 6 hours were harvested after trypsin treatment, resuspended in fresh medium, and plated onto the layer of Matrigel at a density of 1 × 10^5^ cells/well. Culture medium of HCC cells was added and the matrigel cultures were incubated at 37°C. After 8 hours, images (200×) were captured using an Olympus FV1000 Confocal Laser Scanning Microscopy and quantified using Image-J software.

**Aortic ring assay**

Aortic ring assay was assayed as previously described([4](#_ENREF_4)). Aortas were harvested from 7-week-old C57BL/6 normal mice and sectioned into several segments (aortic rings). Plates (48-well) were coated with 120 μL Matrigel. After gelling, the rings were placed into the wells and cultured in DMEM medium containing 1% FBS at 37°C. After 3 days, 200 μL of the culture medium of the differently treated HCC cells was added as indicated. As control, aortic rings were incubated in DMEM medium. The plates were incubated at 37°C, and medium were changed every 2 days for 1 week. The angiogenic sprouting from aortic rings was examined in 3 rings per group (n=3). Each aortic ring was photographed, and sprouting was quantified by counting the number of vascular sprouts that directly originated from the mouse aorta. Photographed image was divided into 2 regions and spouts in each region were scored from 0 (least positive) to 5 (most positive) in a double-blind manner. Each data point took the average and analyzed for vessel spouting([5](#_ENREF_5)).

**RNA-Seq and data analysis**

Sequencing was performed at [BGI](file:////C:\Program%2520Files%2520(x86)\Youdao\Dict\7.5.2.0\resultui\dict\%3fkeyword=BGI)([The](file:////C:\Program%2520Files%2520(x86)\Youdao\Dict\7.5.2.0\resultui\dict\%3fkeyword=The)[Beijing](file:////C:\Program%2520Files%2520(x86)\Youdao\Dict\7.5.2.0\resultui\dict\%3fkeyword=Beijing)[Genomics](file:////C:\Program%2520Files%2520(x86)\Youdao\Dict\7.5.2.0\resultui\dict\%3fkeyword=Genomics)[Institute](file:////C:\Program%2520Files%2520(x86)\Youdao\Dict\7.5.2.0\resultui\dict\%3fkeyword=Institute)) using the BGISEQ-500 platform. RNAseq data was aligned to the reference human genome and transcriptome using HISAT (Hierarchical Indexing for Spliced Alignment of Transcripts). RSEM (RNAseq by Expectation- Maximization) is applied to estimate gene expression levels from RNAseq data. Gene functional hallmark enrichment analysis was performed using GSEA (<http://software.broadinstitute.org/gsea/index.jsp>). The heat map of count data (log_2_(FPKM+1)) was performed using MultiExperiment Viewer (MeV 4.0).

**Statistical analysis**

All experiments were repeated three times. The data were described as mean ± standard deviation (SD). The differences between groups were analyzed by One-Way ANOVA, Dunnett, Student’s t-test or simple [correlation](file:////C:\Program%2520Files%2520(x86)\Youdao\Dict\7.0.1.0227\resultui\dict\%3fkeyword=correlation) [analysis](file:////C:\Program%2520Files%2520(x86)\Youdao\Dict\7.0.1.0227\resultui\dict\%3fkeyword=analysis) with SPSS 22.0 according to data feature. Significant level is *p* < 0.05.

**Reference**

1. Huang Z, Zhou X, He Y, Ke X, Wen Y, Zou F, et al. Hyperthermia enhances 17-DMAG efficacy in hepatocellular carcinoma cells with aggravated DNA damage and impaired G2/M transition. Scientific reports. 2016;6:38072.

2. Nelson JD, Oleg D, Pavel S, Karol B. Fast chromatin immunoprecipitation assay. Nucleic acids research. 2006;34(1):e2.

3. Arnaoutova I, Kleinman HK. In vitro angiogenesis: endothelial cell tube formation on gelled basement membrane extract. Nature Protocols. 2010;5(4):628-35.

4. Baker M, Robinson SD, Lechertier T, Barber PR, Tavora B, D'Amico G, et al. Use of the mouse aortic ring assay to study angiogenesis. Nature Protocols. 2012;7(1):89.

5. YS C, HJ C, JK M, BJ P, YS M, H P, et al. Interleukin-33 induces angiogenesis and vascular permeability through ST2/TRAF6-mediated endothelial nitric oxide production. Blood. 2009;114(14):3117-26.

**Supplementary figure legends**

**Figure S1. Bclaf1 RS domain regulates transcription of HIF1A**. **a, b** Bclaf1 and HIF-1α protein (a) and mRNA (b) levels in transfected Huh7 and HepG2 cells. **c, d** Huh7 and HepG2 cells were transfected with the indicated plasmids, incubated for 24 hours and then cultured under hypoxic condition (1% O_2_) for 24 hours. The BCLAF1 and HIF1A mRNA expression levels were assessed with RT-qPCR. **e** ChIP analysis of the HIF1A promoter in Huh7 cells exposed to hypoxia for 24h after transfection. Immunoprecipitation was performed using anti-Bclaf1 and control IgG antibodies followed by qPCR analysis. Data represent mean ± SD of three independent experiments. **p*<0.05, ***p*<0.01, ****p*<0.001.

**Figure S2. HIF-1α and its downstream angiogenesis genes are targets of Bclaf1**. Huh7 and HepG2 cells were transfected with siRNA or plasmids for 24 hours and subsequently cultured in hypoxia (1% O_2_) for 24 hours. **a, d** The mRNA levels were assessed with RT-qPCR. **b, c** The HIF-1α protein (b) and mRNA (c) levels in transfected HCC cells. Data represent mean ± SD of three independent experiments. **p*<0.05, ***p*<0.01, ****p*<0.001.

**Figure S3. Bclaf1 does not affect HIF1A mRNA translation or HIF-1α degradation**. Huh7 cells were transfected with siRNA for 24 hours and subsequently cultured in hypoxia (1% O_2_) for 24 hours. Before sample collected, cells were treated with the proteasome inhibitor MG132 (30mM, 30min) or the protein synthesis inhibitor cycloheximide (CHX: 125μg/mL, 0, 1, 2, 4 hours) for the indicated time. Bclaf1 and HIF-1α protein amounts were assessed by Western blot analysis (**a, b**). Data represent mean ± SD of three independent experiments. **p*<0.05, ***p*<0.01.

**Figure S4. c-Myc has no significant effect on HIF1A transcription**. Huh7 and HepG2 cells were transfected with siRNA for 24 hours and subsequently cultured in hypoxia (1% O_2_) for 24 hours. **a** The mRNA expression levels were assessed with RT-qPCR. **b** mRNA levels were analyzed by RT-PCR and Gel-Pro software. Data represent mean ± SD of three independent experiments. NS, not significant, ***p*<0.01, ****p*<0.001.

**Figure S5. HIF1A silencing reduces Bclaf1 protein amounts in hypoxic conditions**. HepG2 and Huh7 cells were transfected with siNC (negative control) or siHIF1A for 24 hours and subsequently cultured in hypoxia (1% O_2_) for 24 hours. The Bclaf1 amounts were assessed by Western blot analysis (**a**) and Immunofluorescence staining (**b**).
