# Supplementary figures and images for: Bclaf1 promotes angiogenesis by regulating HIF-1α transcription in hepatocellular carcinoma
Source: Oncogene. 2018 Oct 26;38(11):1845–59. doi: 10.1038/s41388-018-0552-1 (PMC6462866; doi:10.1038/s41388-018-0552-1)

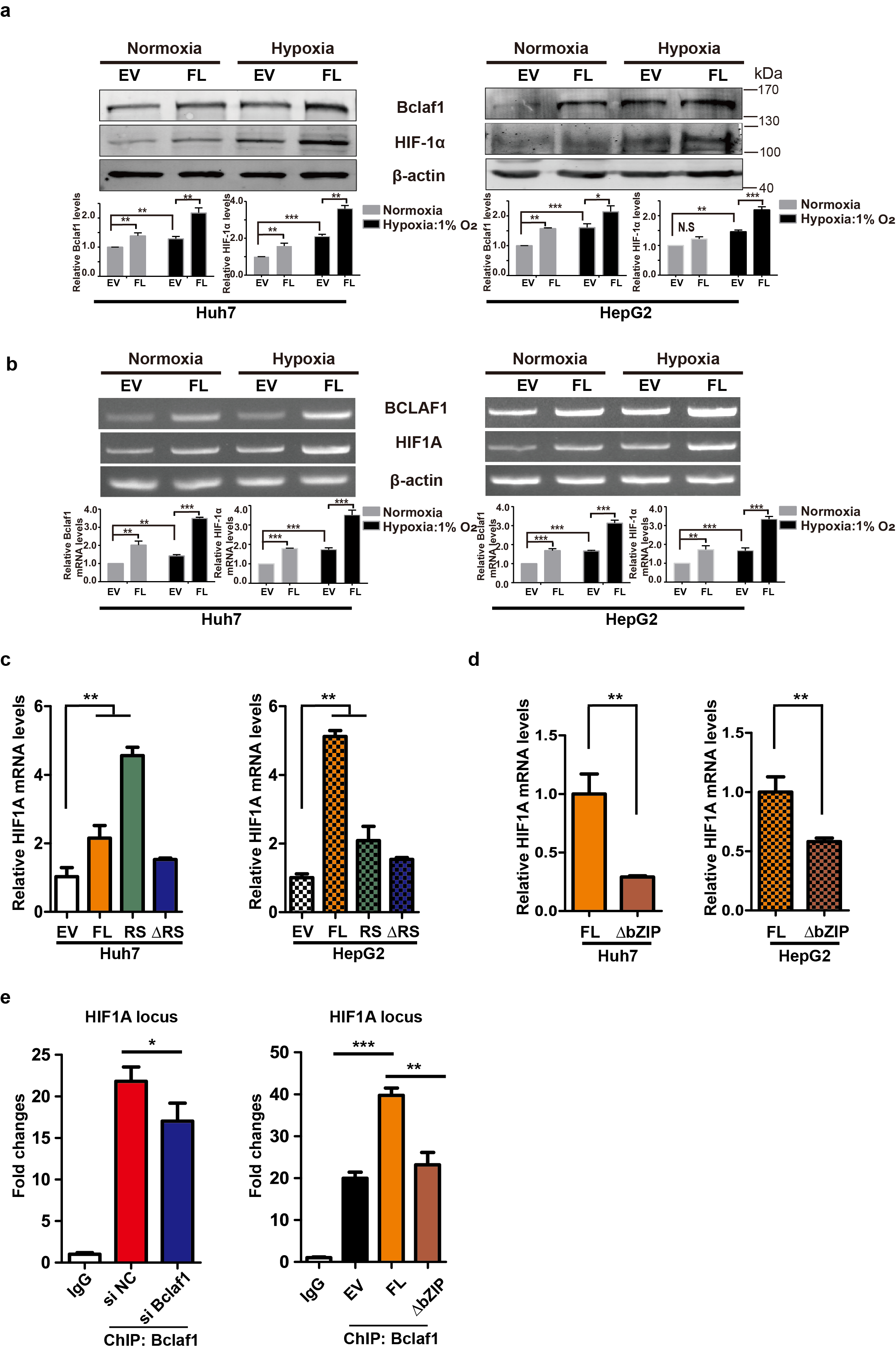

Supplement: Supplementary file 2 — Fig.S1 [file 41388_2018_552_MOESM2_ESM.tif]

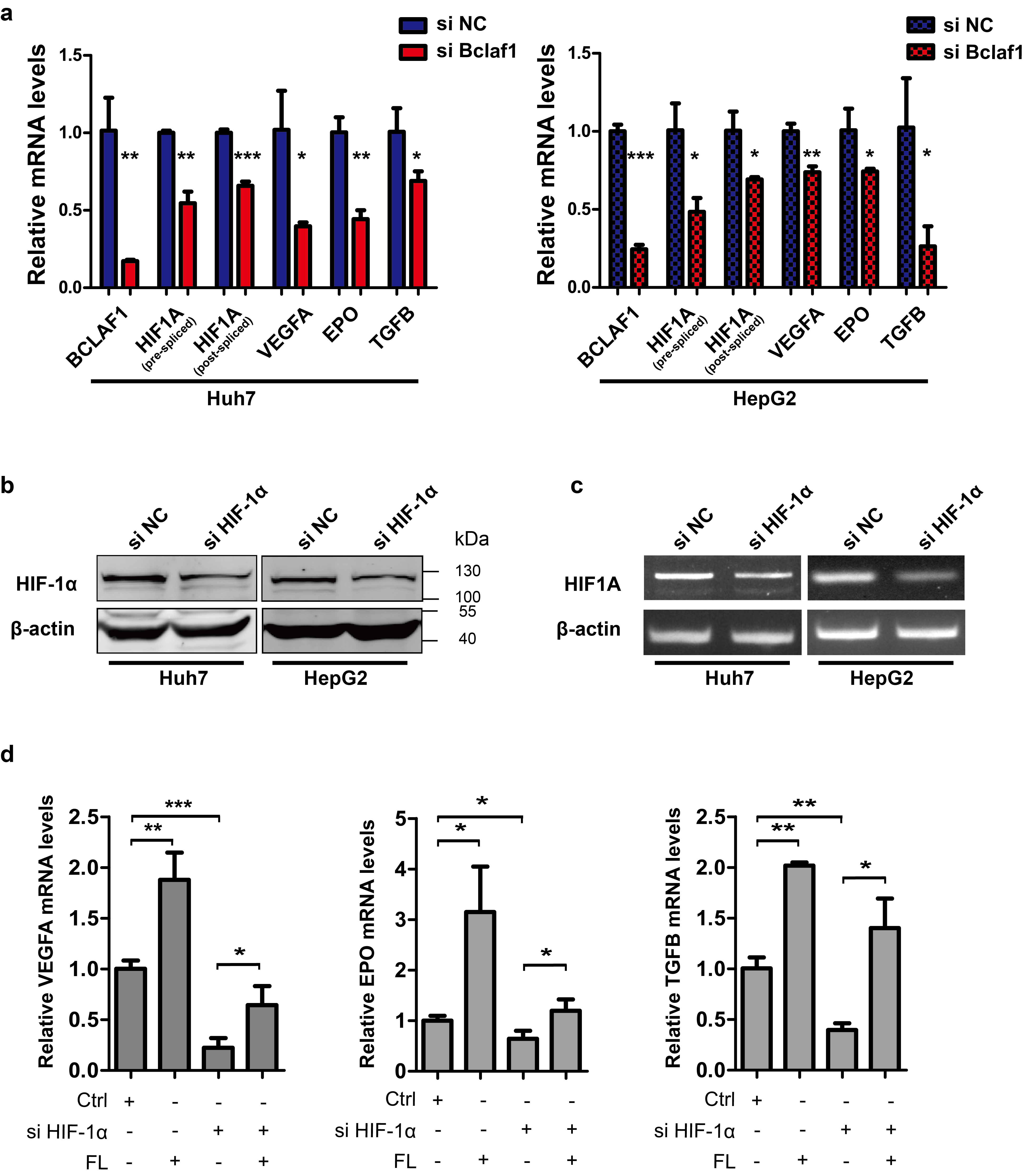

Supplement: Supplementary file 3 — Fig.S2 [file 41388_2018_552_MOESM3_ESM.tif]

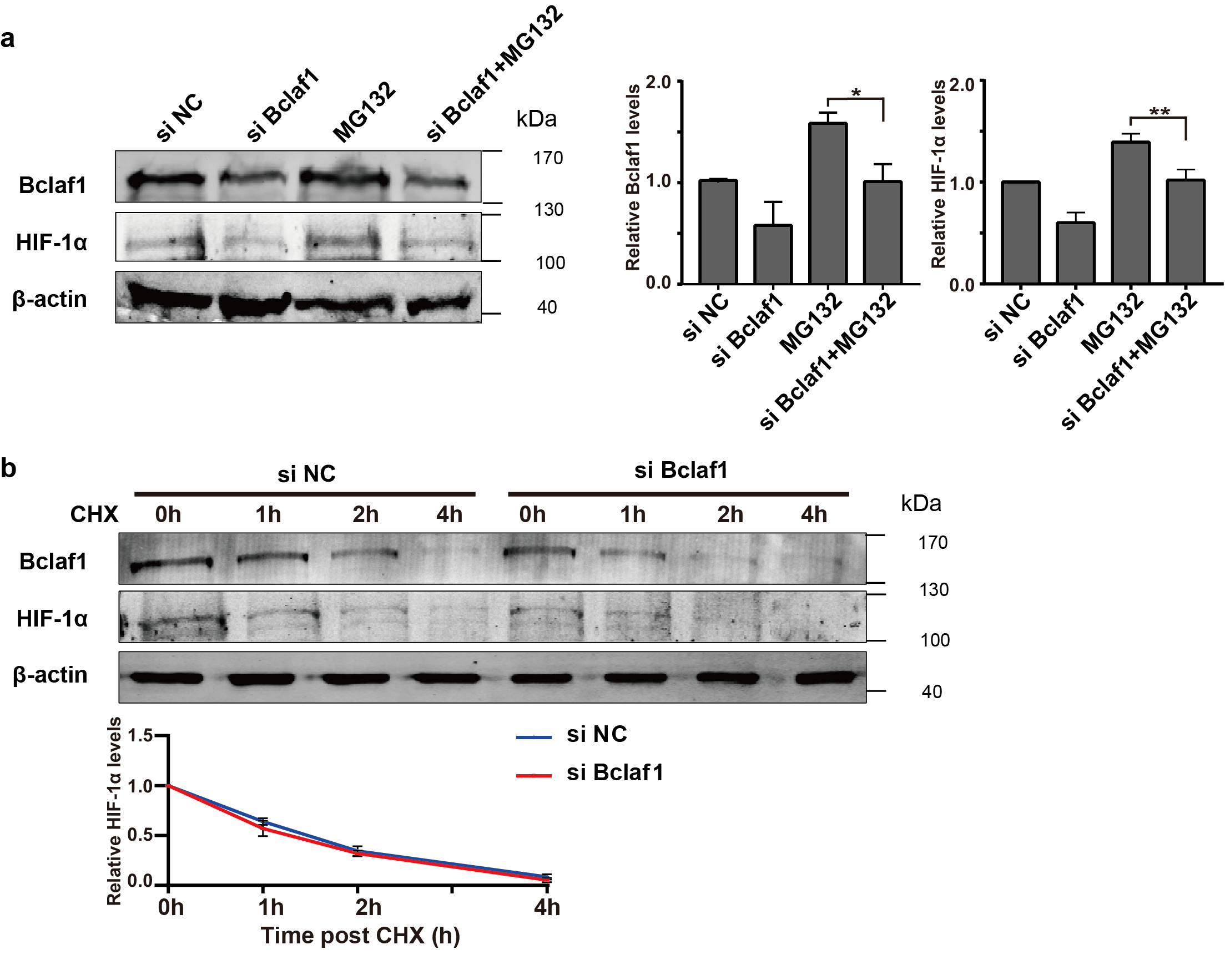

Supplement: Supplementary file 4 — Fig.S3 [file 41388_2018_552_MOESM4_ESM.tif]

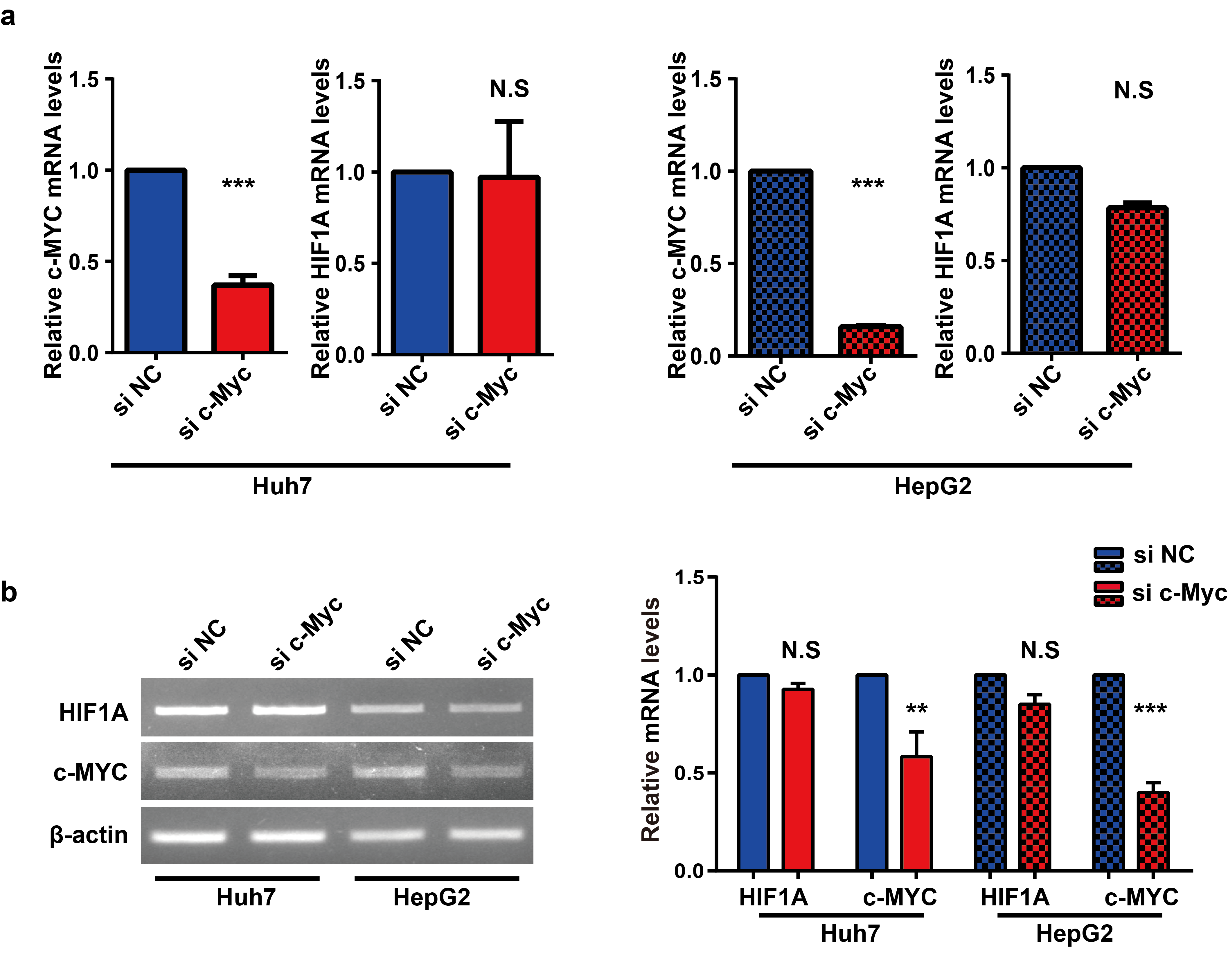

Supplement: Supplementary file 5 — Fig.S4 [file 41388_2018_552_MOESM5_ESM.tif]

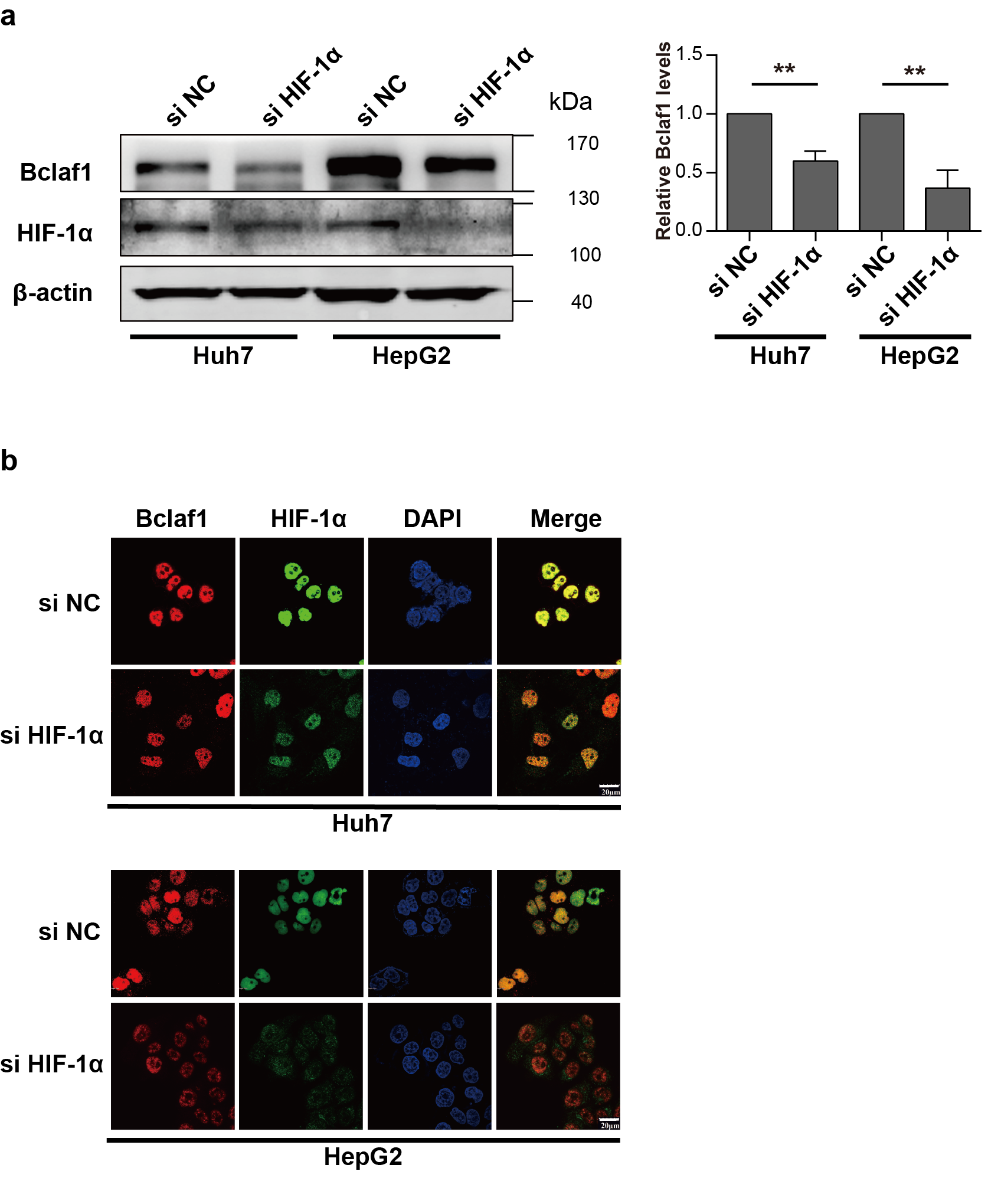

Supplement: Supplementary file 6 — Fig.S5 [file 41388_2018_552_MOESM6_ESM.tif]
