# Supplementary material for: Bclaf1 promotes angiogenesis by regulating HIF-1α transcription in hepatocellular carcinoma
Source: Oncogene. 2018 Oct 26;38(11):1845–59. doi: 10.1038/s41388-018-0552-1 (PMC6462866; doi:10.1038/s41388-018-0552-1)
Supplement: Supplementary file 7 — Tab.S1 [file 41388_2018_552_MOESM7_ESM.docx]

**Table S1. Clinicopathological characteristics of 15 hepatocarcinomapatients.**

| Pathological  lD | Gender | Age | Tumor size  (cm*cm*cm) | Procedure | Edmondson  grade | TNM Stage | Cirrhosis | HBV |
| --- | --- | --- | --- | --- | --- | --- | --- | --- |
| 436097 | Male | 56 | 5*5*8 | surgery | Ⅰ | T2 N0 M0 | Yes | Positive |
| 436436 | Male | 62 | 6*3*1.5 | surgery | Ⅱ | T2 N0 M0 | No | Positive |
| 436648 | Male | 45 | 4*3*5 | surgery | Ⅰ | T2 N0 M0 | Yes | Positive |
| 440257 | Male | 39 | 2*3*2 | surgery | Ⅱ | T2 N0 M0 | No | Positive |
| 447092 | Male | 62 | 4.5*5*3 | surgery | Ⅰ | T2 N0 M0 | No | Positive |
| 448544 | Female | 43 | 4*2*2 | surgery | Ⅰ | T2 N0 M0 | No | Negative |
| 457926 | Male | 43 | 5*1.5*3 | surgery | Ⅱ | T2 N0 M0 | No | Positive |
| 436473 | Male | 65 | 3*3.5*4 | surgery | Ⅰ | T2 N0 M0 | Yes | Negative |
| 449753 | Female | 49 | 5*5*6 | surgery | Ⅱ | T2 N0 M0 | No | Negative |
| 458663 | Female | 51 | 2.5*2.5*3 | surgery | Ⅱ | T2 N0 M0 | Yes | Positive |
| 464032 | Male | 61 | 6*5*2 | surgery | Ⅰ | T2 N0 M0 | Yes | Negative |
| 468953 | Male | 46 | 3*5*2.5 | surgery | Ⅱ | T2 N0 M0 | No | Negative |
| 463094 | Male | 58 | 3*1.5*2 | surgery | Ⅱ | T2 N0 M0 | No | Negative |
| 475699 | Male | 60 | 4*2*2 | surgery | Ⅱ | T2 N0 M0 | Yes | Positive |
| 476377 | Female | 54 | 3*6*3 | surgery | Ⅱ | T2 N0 M0 | Yes | Negative |

**Table S2. The primers of gene sequences list**

| GENE | PCR primer | |
| --- | --- | --- |
| BCLAF1 | | F 5′- TCTGGAATAGAAGGCACTCTAGG-3′  R 5′- ACCCTCGTCTTTTAGAAACAGGA-3′ |
| HIF1A (post-spliced) | | F 5′- CAAGAACCTACTGCTAATGC-3′  R 5′- GTCTGGCTGCTGTAATAATG-3 |
| HIF1A (pre-spliced) | | F 5′- CAGCTATTTGCGTGTGAGGA-3′  R 5′- AACATTGCGACCACCTTCTAA-3′ |
| HIF1A (P1) | | F 5′- ATTATCCGAGTGTGGTGGTG-3′  R 5′- GAGGTGTGAGGCTGGAGAAG-3′ |
| HIF1A (P2) | | F 5′- CTTCTCCAGCCTCACACCTC-3′  R 5′- CCTAACCATGGGTCAATGTCA-3′ |
| HIF1A (P3) | | F 5′- TGACATTGACCCATGGTTAGG-3′  R 5′- CTGTGCACTGAGGAGCTGAG-3′ |
| β-actin | | F 5′-CATGTACGTTGCTATCCAGGC-3′  R 5′-CTCCTTAATGTCACGCACGAT-3′ |
| VEGFA | | \| R 5′- AACCATGAACTTTCTGCTGT-3′ \| \| --- \| \| R 5′- GGCTTGAAGATGTACTCGAT-3′ \| |
| EPO | | \| R 5′- ATGTGGATAAAGCCGTCAG-3′ \| \| --- \| \| R 5′- ATTGGAGTAGACTCGGAAGA-3′ \| |
| TGFB | | \| R 5′- TATTTAAGGACACCCGTGC-3′ \| \| --- \| \| R 5′- CAATGACACAGAGATCCGC-3′ \| |
| c-MYC | | F 5′-TGGAACTTACAACACCCG-3′ |
|  |  | R 5′-CCTCGTCGCAGTAGAAAT-3′ |

**Table S3. The amplification curves and melting peaks of primer pairs.**

**BCLAF1**

**β-actin**


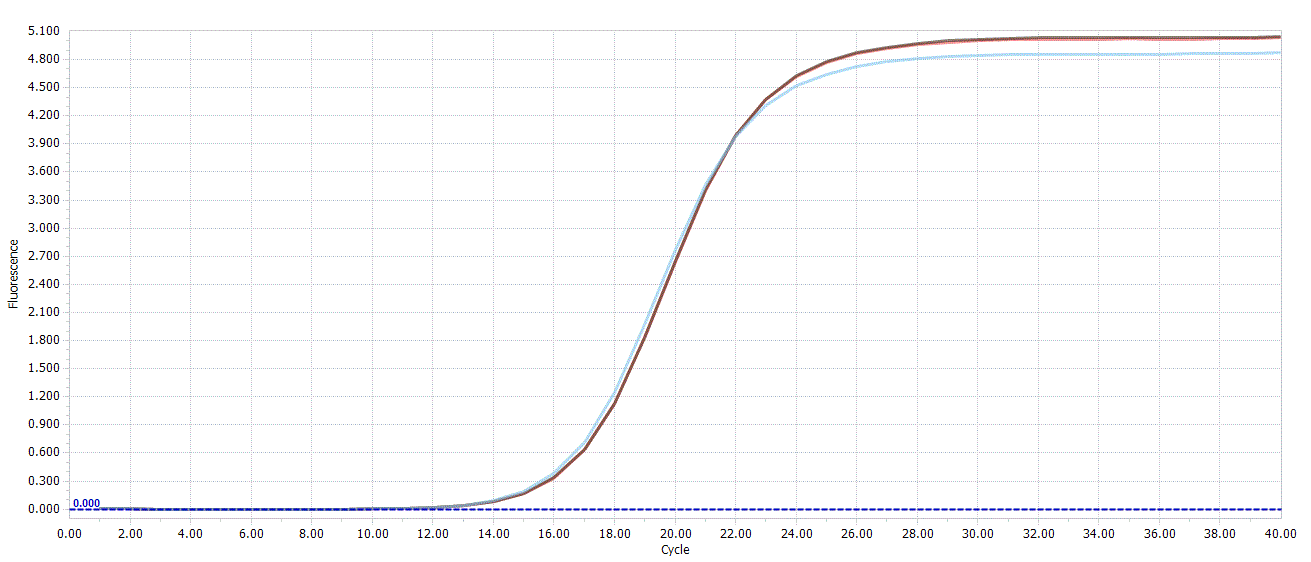

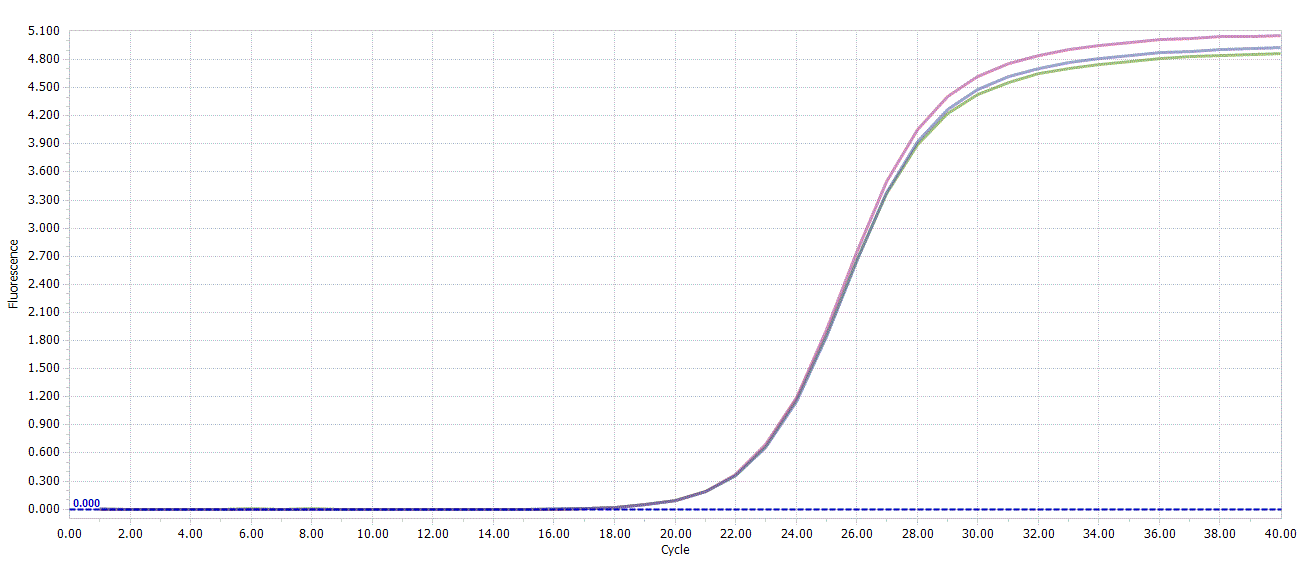


Amplification Curves


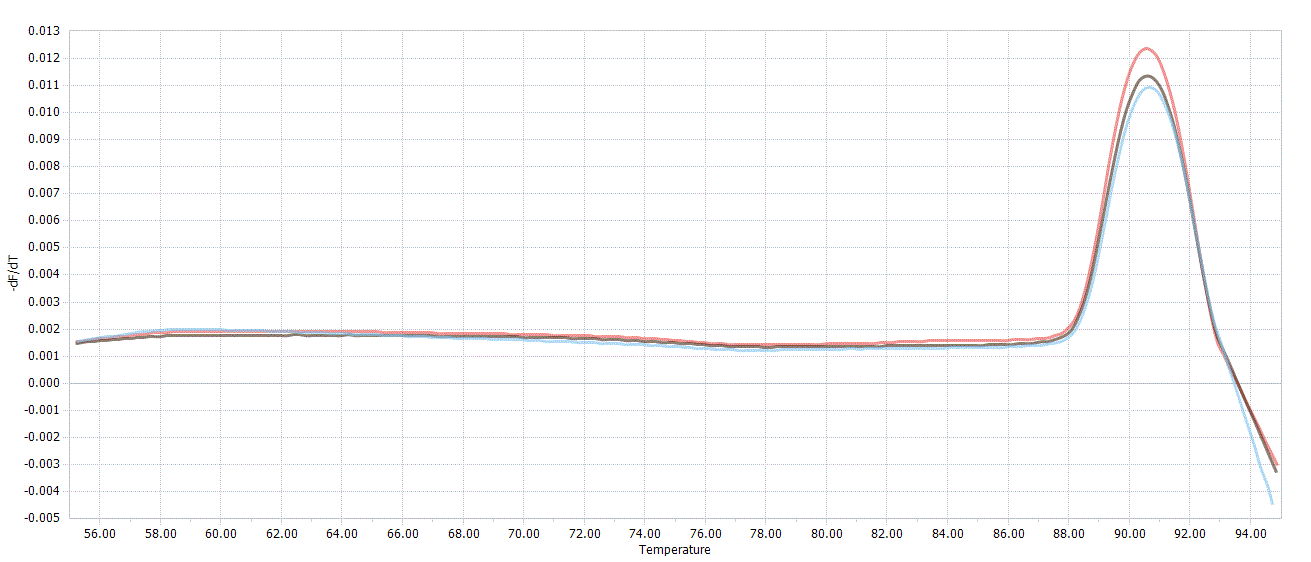

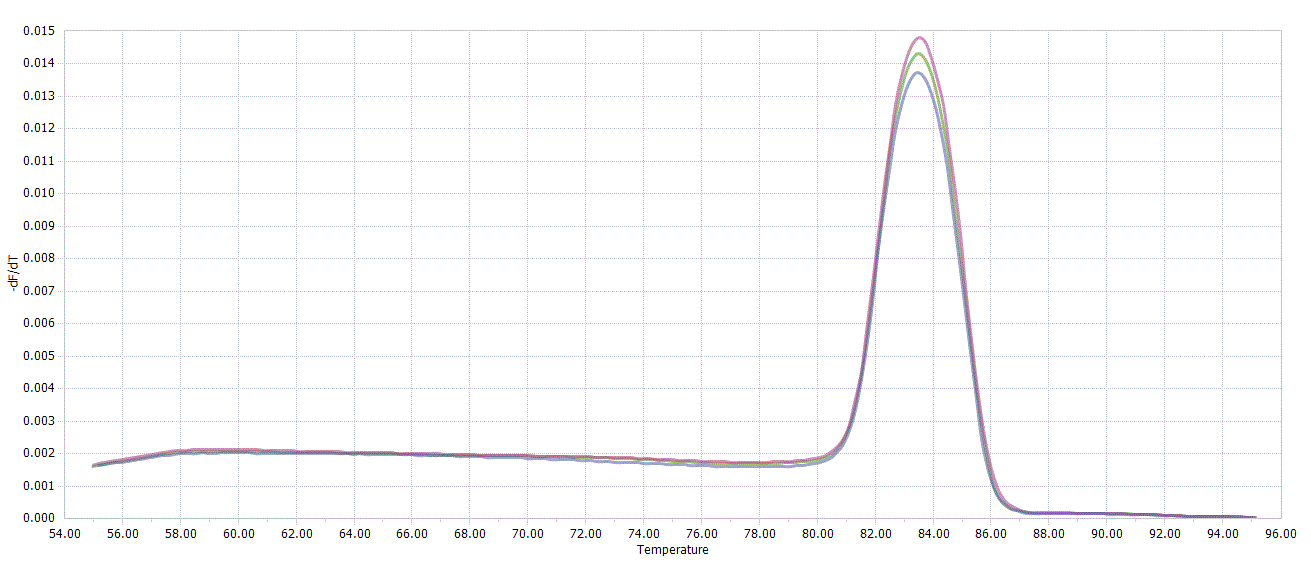


Melting Peaks

**HIF1A (pre-spliced)**

**HIF1A (post-spliced)**


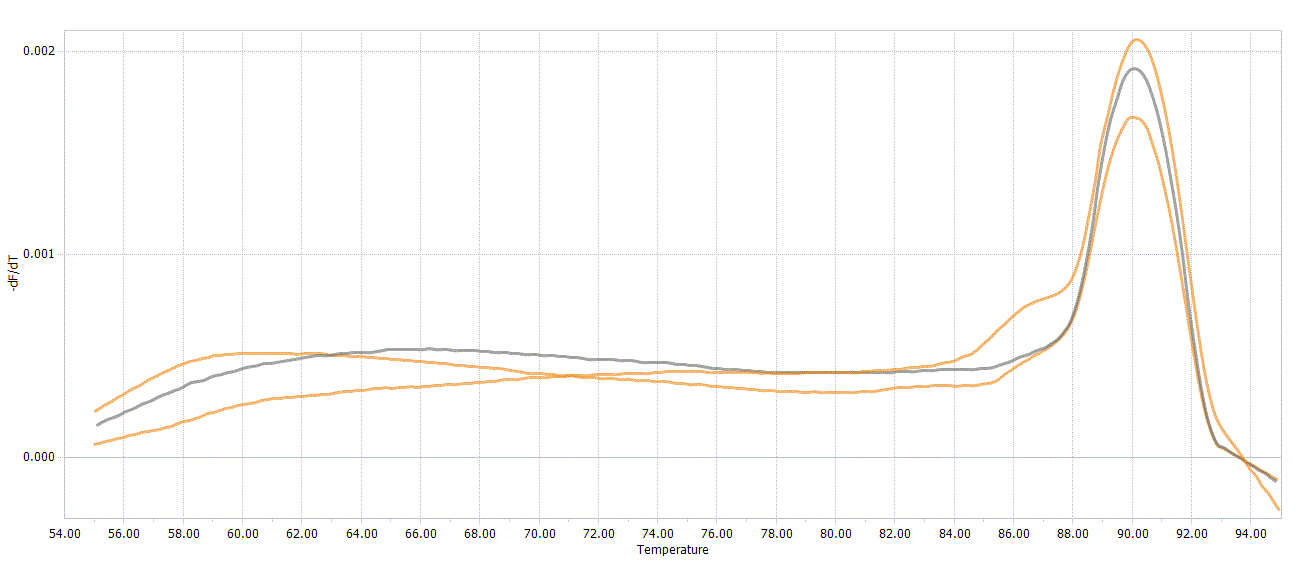

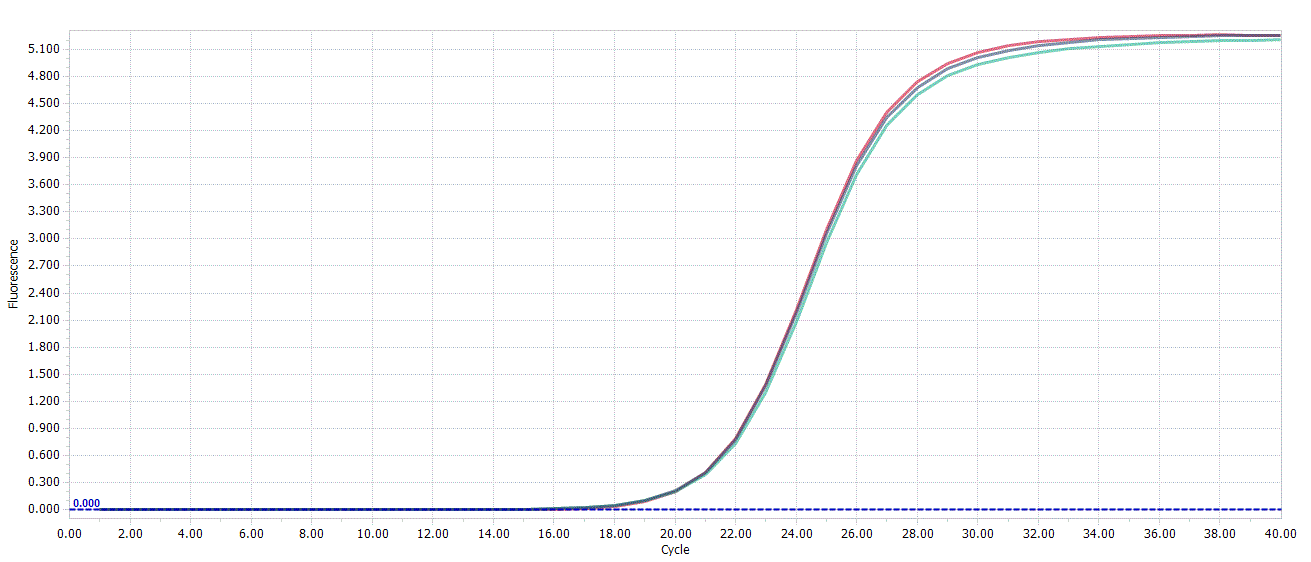


Amplification Curves


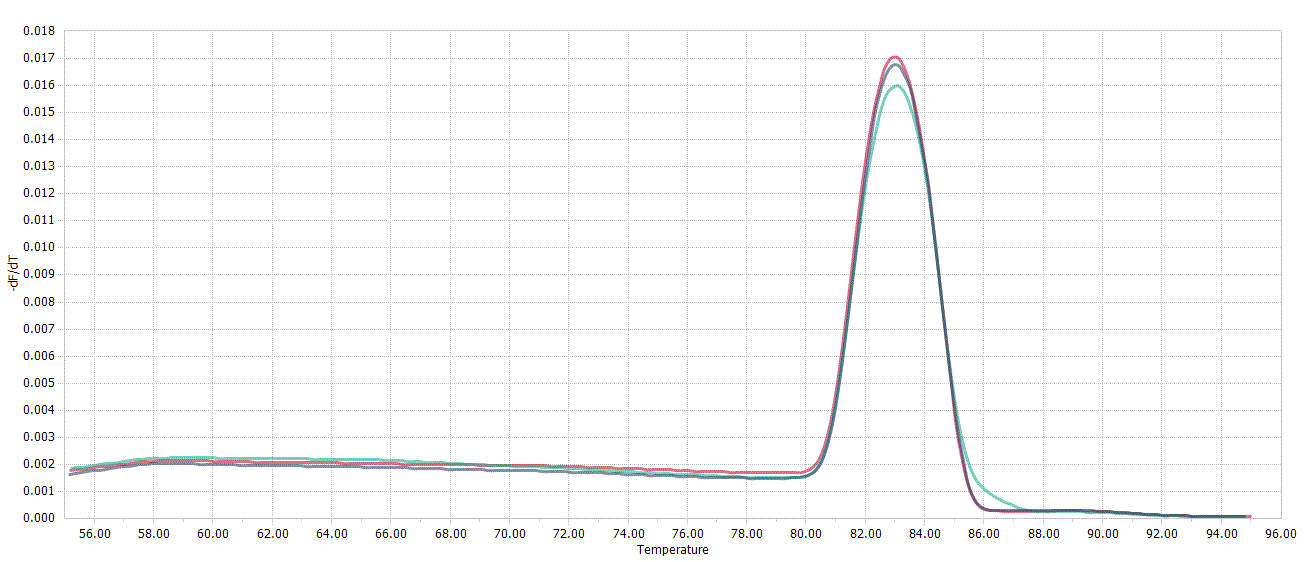

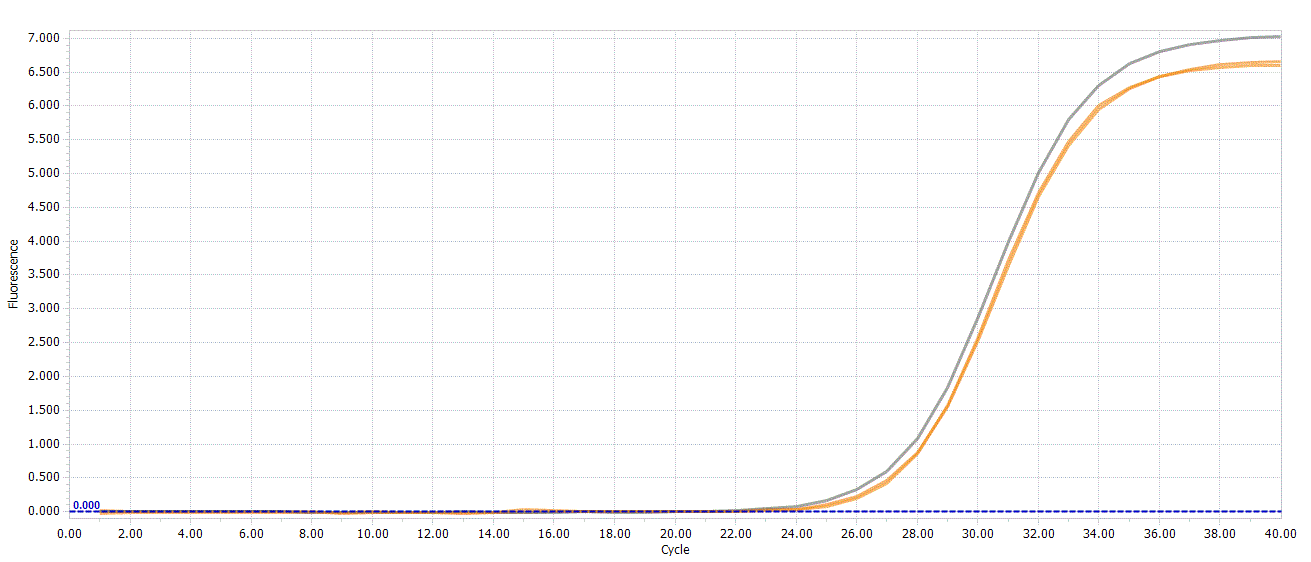


Melting Peaks

**HIF1A (P1)**

**HIF1A (P2)**


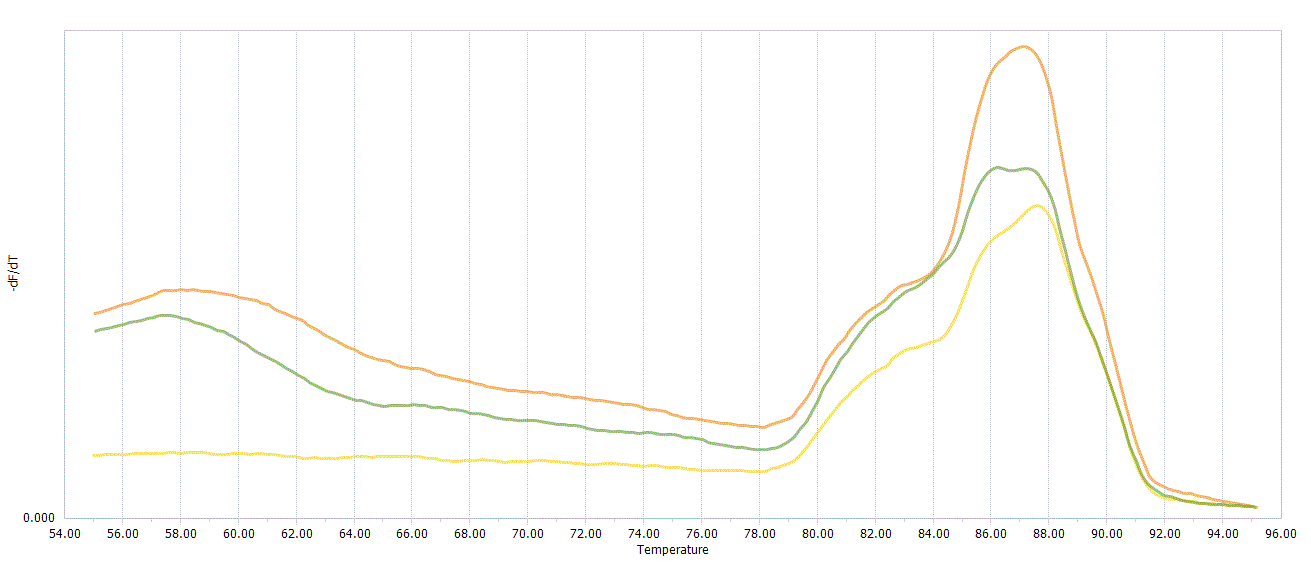

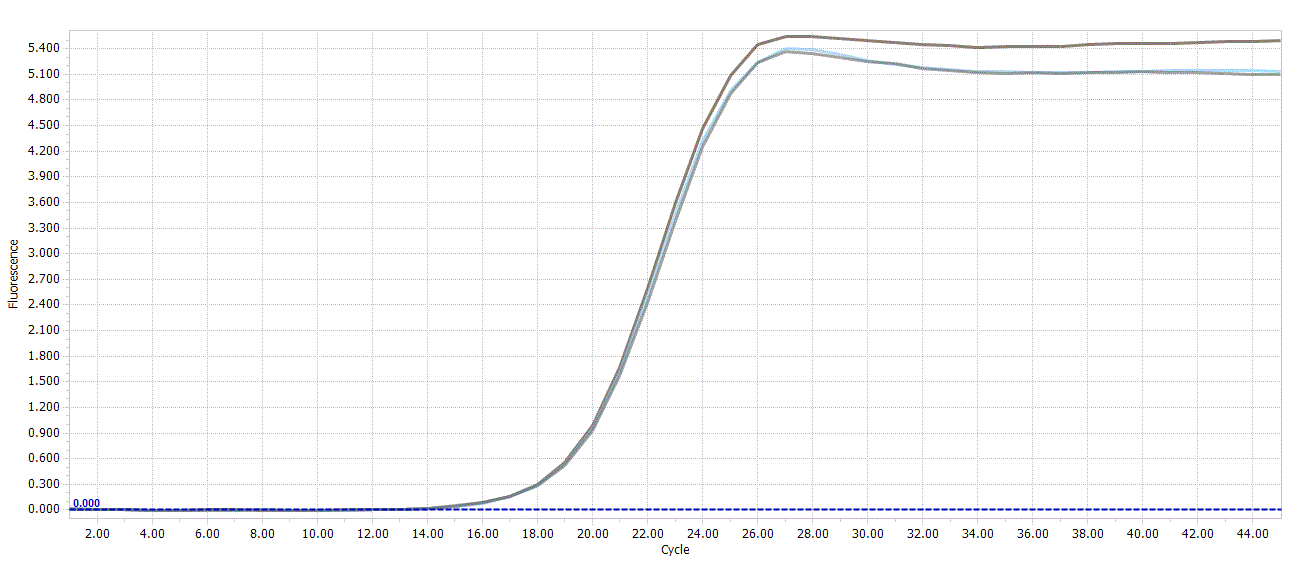


Amplification Curves


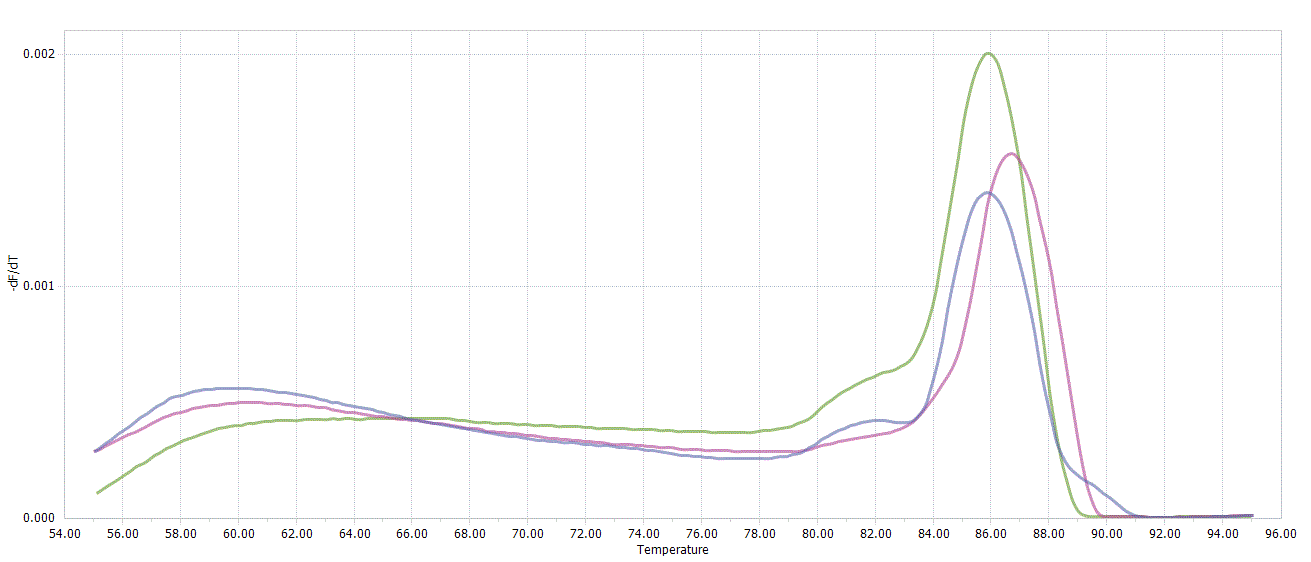

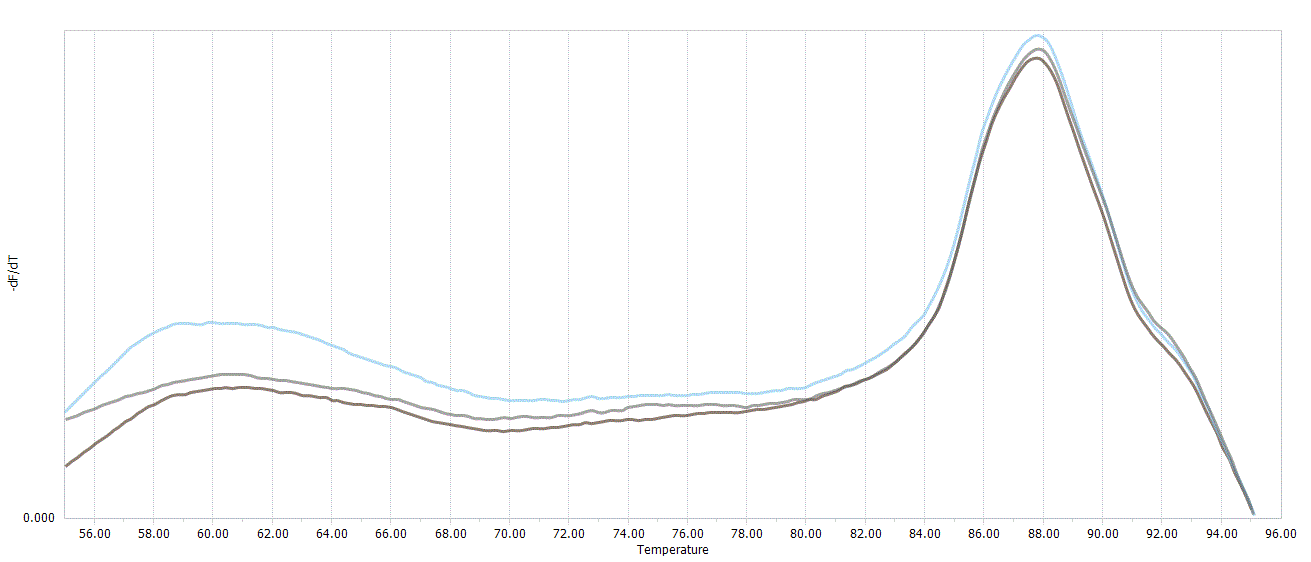


Melting Peaks

**VEGF**

**HIF1A (P3)**


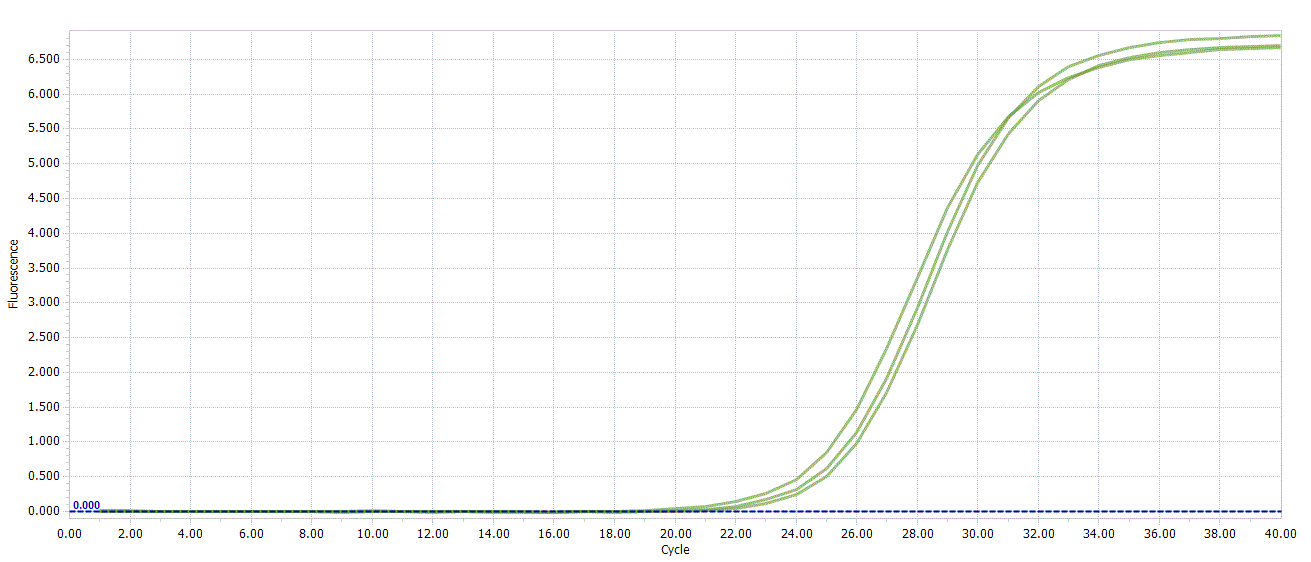

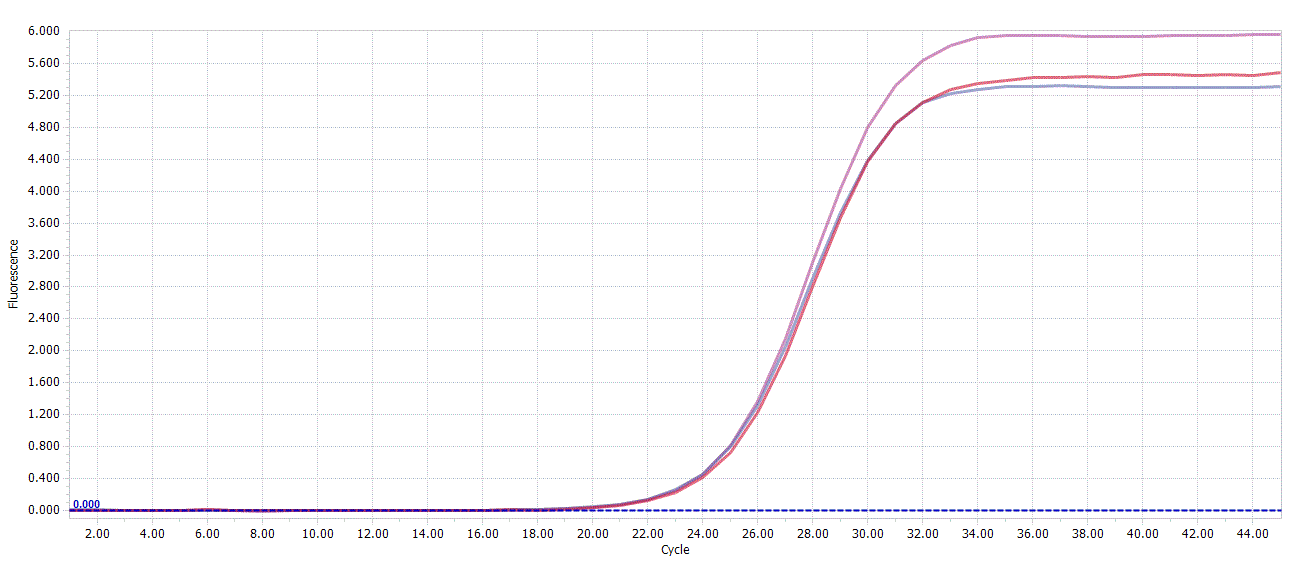


Amplification Curves


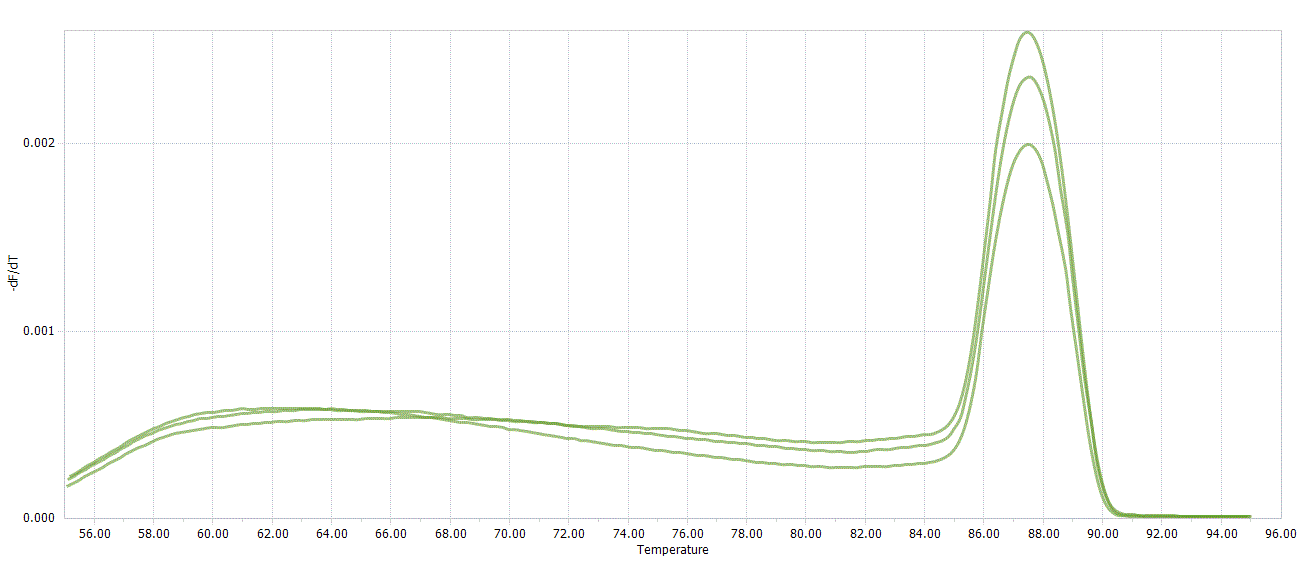

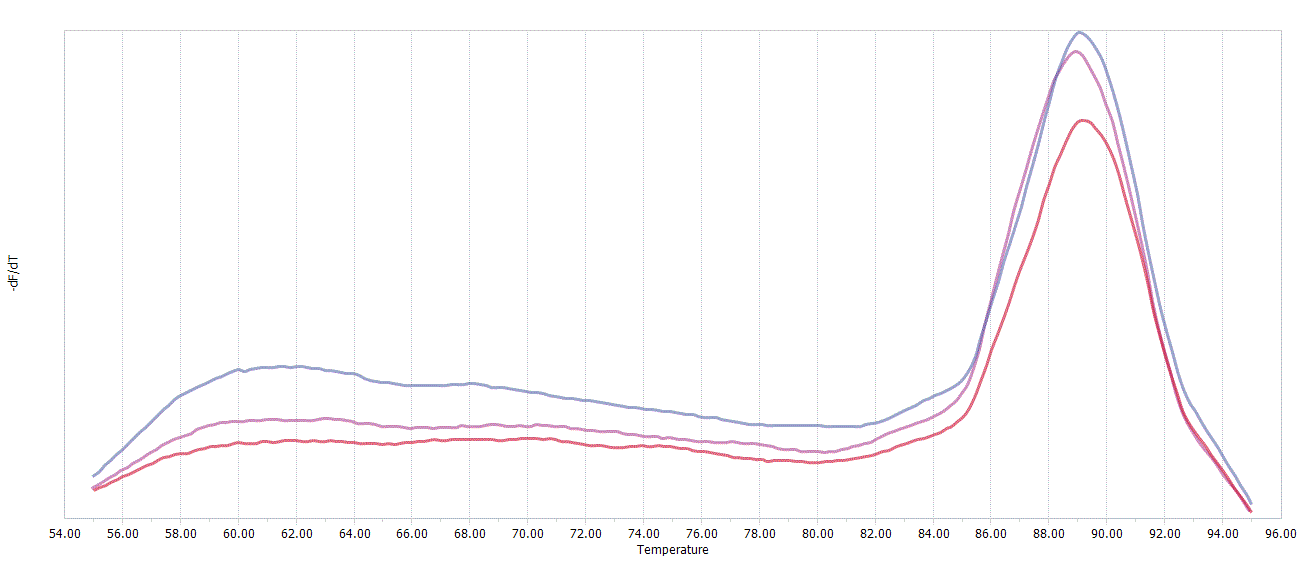


Melting Peaks

**TGFB**

**EPO**


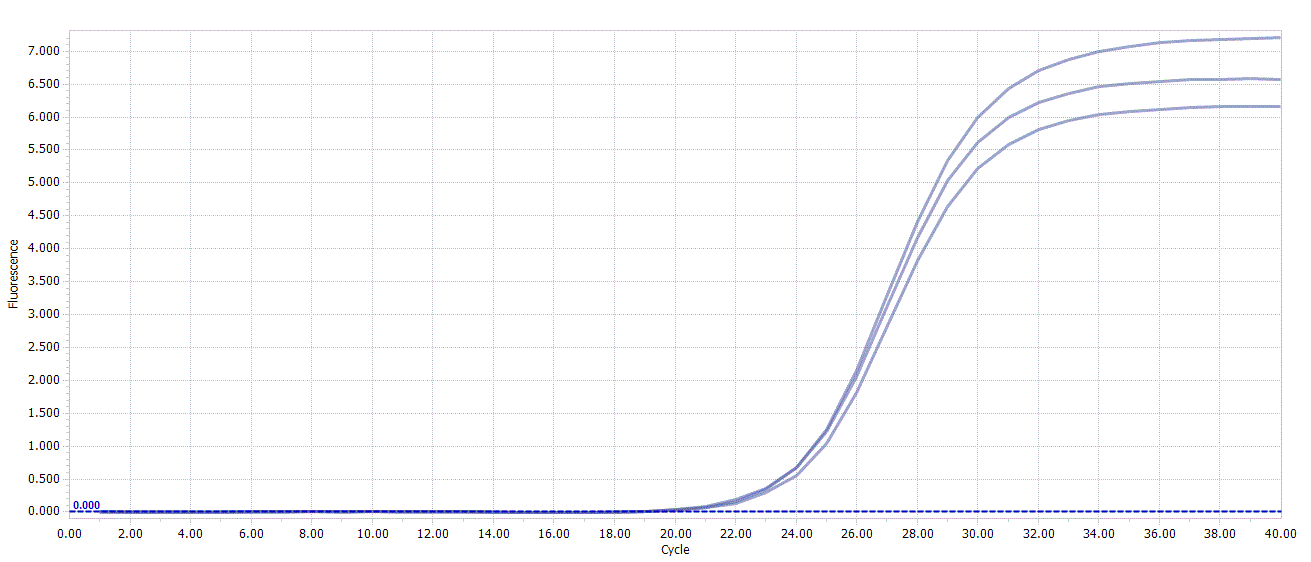

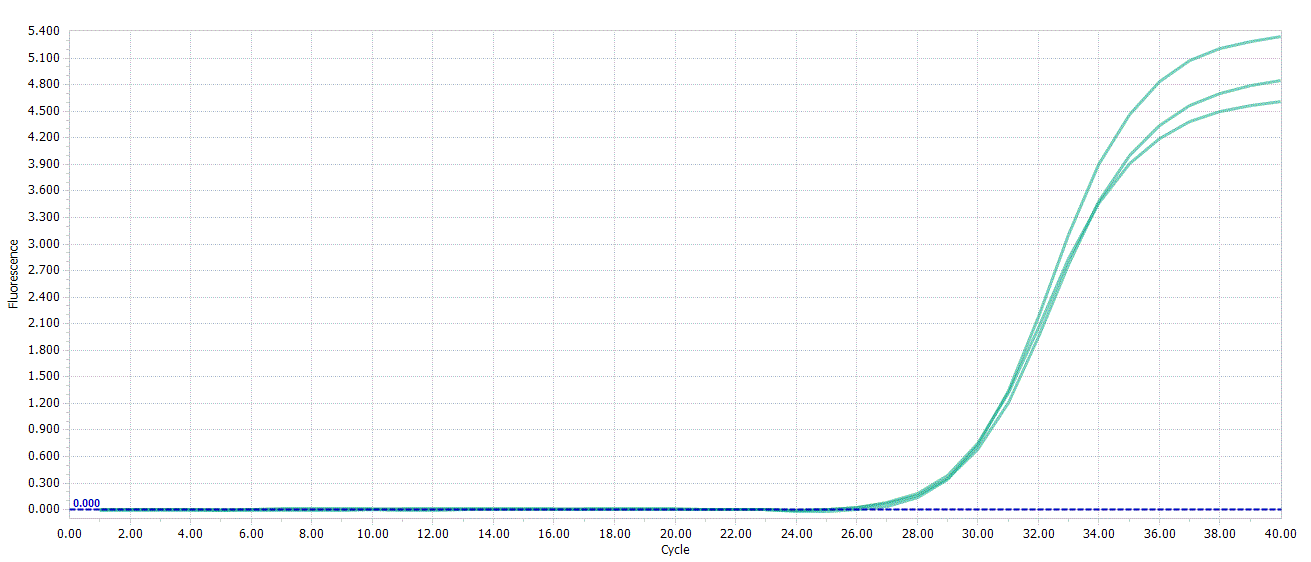


Amplification Curves


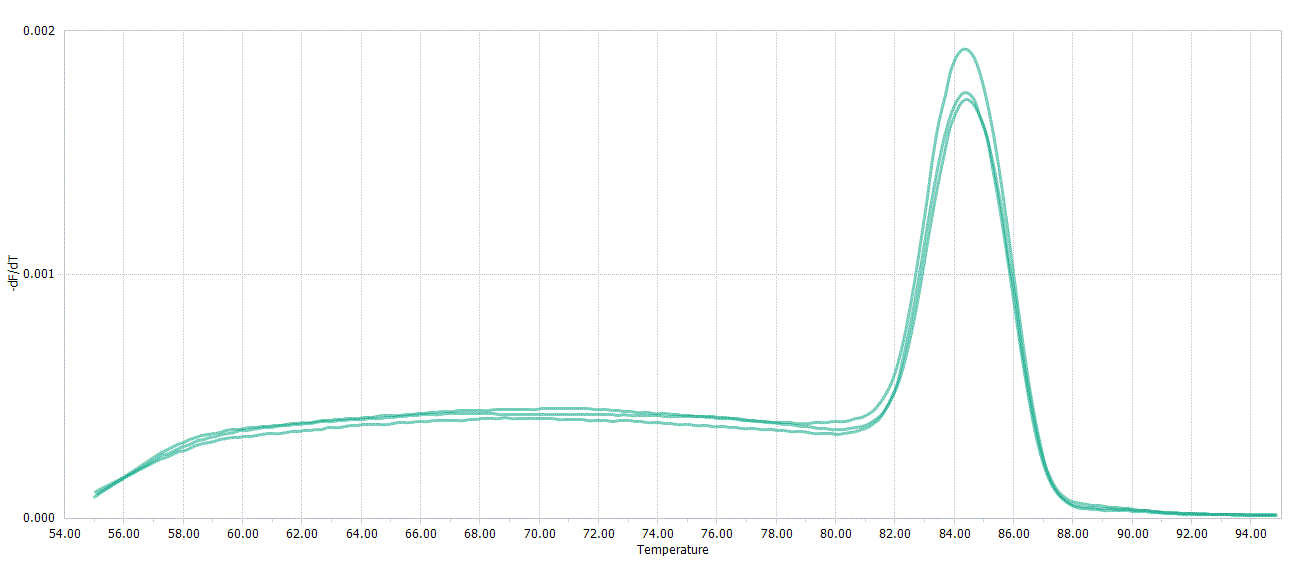

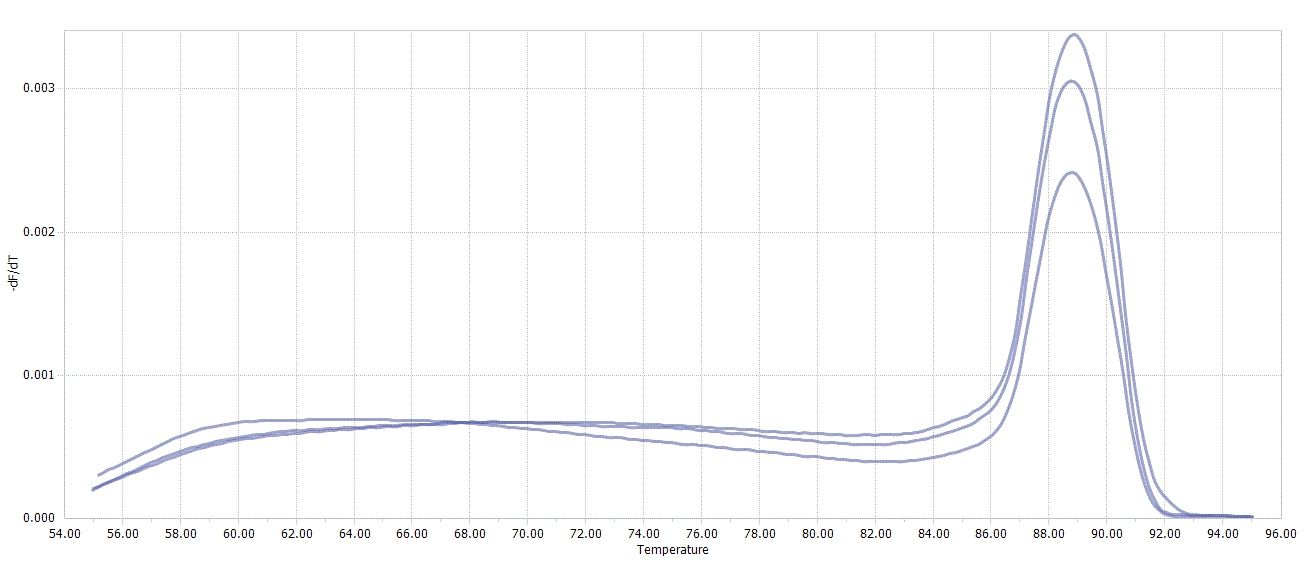


Melting Peaks

**c-MYC**


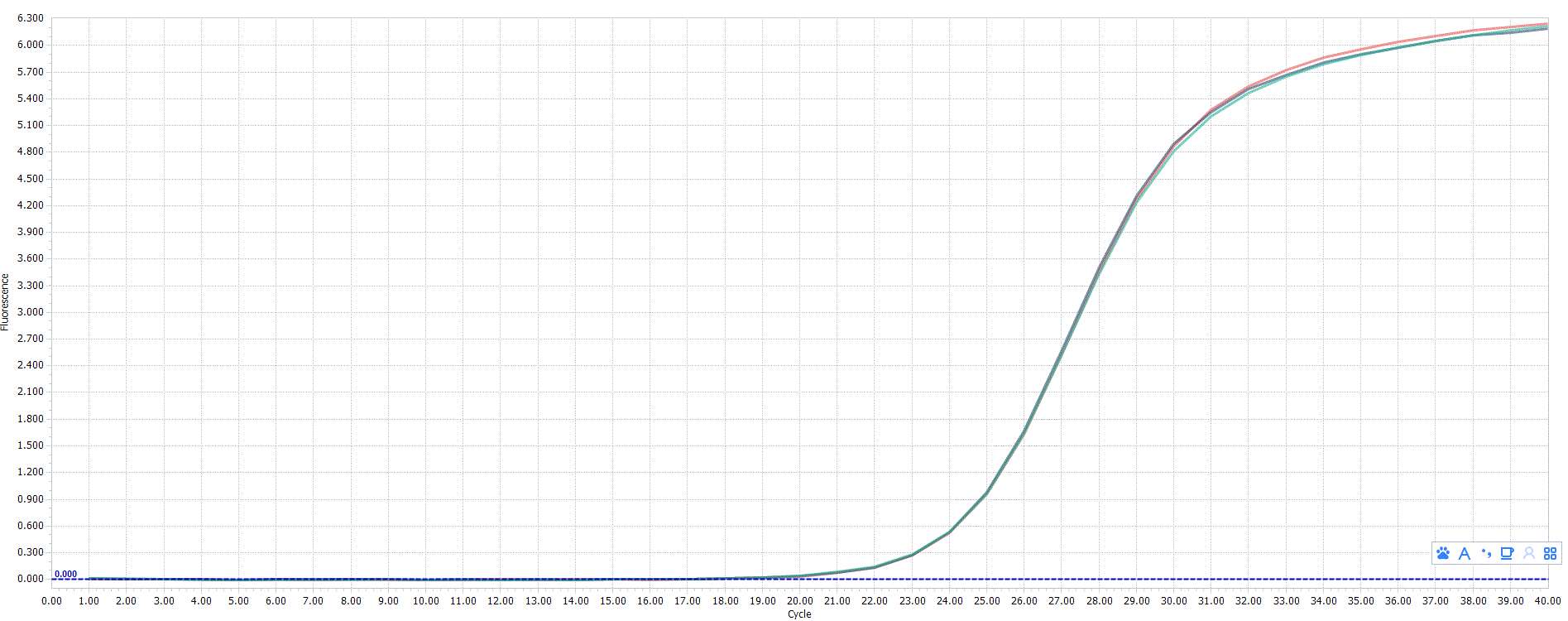


Amplification Curves


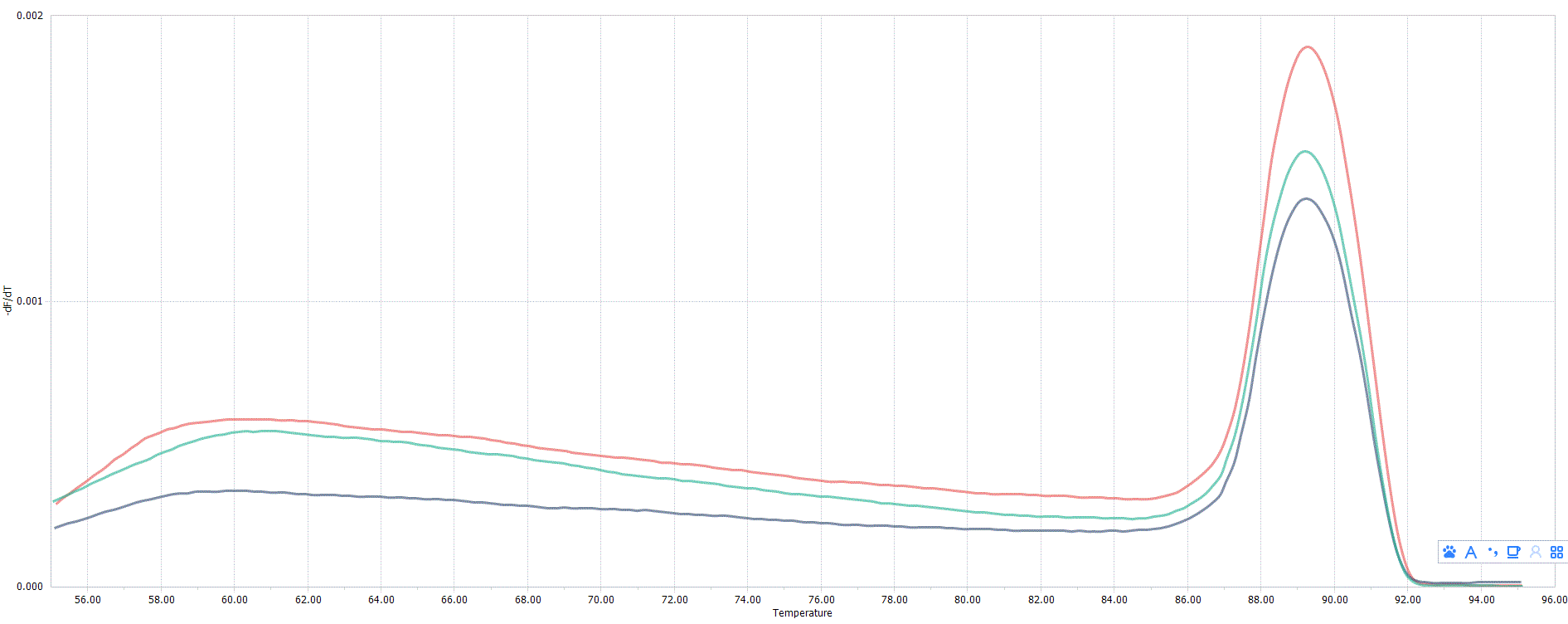


Melting Peaks
